# RNA-driven JAZF1-SUZ12 gene fusion in human endometrial stromal cells

**DOI:** 10.1371/journal.pgen.1009985

**Published:** 2021-12-20

**Authors:** Sachin Kumar Gupta, Jocelyn Duen-Ya Jea, Laising Yen

**Affiliations:** 1 Department of Pathology & Immunology, Baylor College of Medicine, Houston, Texas, United States of America; 2 Department of Molecular & Cellular Biology, Baylor College of Medicine, Houston, Texas, United States of America; 3 Dan L. Duncan Cancer Center, Baylor College of Medicine, Houston, Texas, United States of America; Bar-Ilan University, ISRAEL

## Abstract

Oncogenic fusion genes as the result of chromosomal rearrangements are important for understanding genome instability in cancer cells and developing useful cancer therapies. To date, the mechanisms that create such oncogenic fusion genes are poorly understood. Previously we reported an unappreciated RNA-driven mechanism in human prostate cells in which the expression of chimeric RNA induces specified gene fusions in a sequence-dependent manner. One fundamental question yet to be addressed is whether such RNA-driven gene fusion mechanism is generalizable, or rather, a special case restricted to prostate cells. In this report, we demonstrated that the expression of designed chimeric RNAs in human endometrial stromal cells leads to the formation of JAZF1-SUZ12, a cancer fusion gene commonly found in low-grade endometrial stromal sarcomas. The process is specified by the sequence of chimeric RNA involved and inhibited by estrogen or progesterone. Furthermore, it is the antisense rather than sense chimeric RNAs that effectively drive JAZF1-SUZ12 gene fusion. The induced fusion gene is validated both at the RNA and the genomic DNA level. The ability of designed chimeric RNAs to drive and recapitulate the formation of JAZF1-SUZ12 gene fusion in endometrial cells represents another independent case of RNA-driven gene fusion, suggesting that RNA-driven genomic recombination is a permissible mechanism in mammalian cells. The results could have fundamental implications in the role of RNA in genome stability, and provide important insight in early disease mechanisms related to the formation of cancer fusion genes.

## Author summary

Fusion genes resulting from chromosomal translocations are important for understanding cancer mechanisms and developing anti-cancer therapies. Fusion gene are presumed to occur prior to fusion RNA expression. However, studies have reported the presence of fusion RNAs in individuals who were negative for chromosomal translocations. The observation, that fusion RNA could be present prior to fusion gene formation, raises the provocative hypothesis that fusion RNA, or any cellular RNA with sequence compositions resembling that of fusion RNA, could act as a template to mediate genomic rearrangement which leads to the final gene fusion. In this report, we demonstrated that the expression of designed chimeric RNAs in human endometrial stromal cells leads to the formation of JAZF1-SUZ12, a cancer fusion gene found in endometrial stromal sarcomas. The process is specified by the sequence of chimeric RNA involved and inhibited by estrogen or progesterone. Furthermore, it is the antisense rather than sense chimeric RNAs that effectively drive JAZF1-SUZ12 gene fusion. The results could have fundamental implications in the role of RNA in mammalian genome stability, provide important insight in early disease mechanism, as well as developing gene editing technology via mechanisms native to mammalian cells.

## Introduction

**Fig 1:**
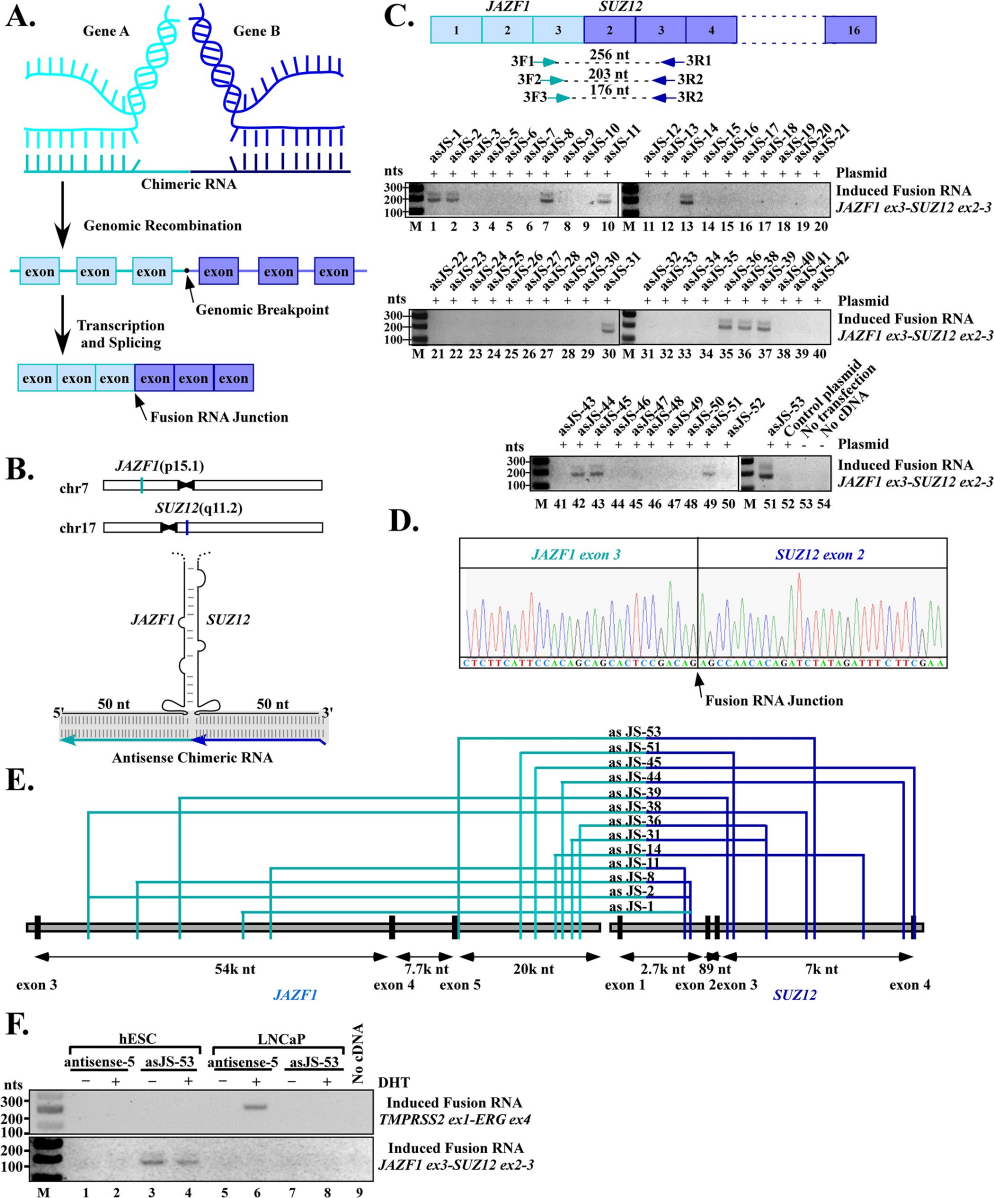
Exogenously expressed chimeric RNAs induce the expression of JAZF1-SUZ12 fusion transcripts found in endometrial stromal sarcoma. (**A)** Model of RNA-mediated gene fusion in mammalian cells. A chimeric RNA invades chromosomal DNA of gene A and gene B in a sequence-dependent manner to stabilize a transient RNA/DNA hybrid. Resolving such an RNA/DNA hybrid by DNA breaks and rearrangements yields the final fusion gene A-B. Transcription of this fusion gene and subsequent RNA splicing produce a fusion RNA consisting of exons from both genes joined together by annotated splice sites. Note that for most known cancer fusion genes, the genomic break points are located in the introns, which is different from fusion RNA junction that is a splice junction. **(B)** Upper panel: chromosomal locations of JAZF1 and SUZ12 genes. Lower panel: schematics of the putative three-way junctions formed among JAZF1 and SUZ12 genomic DNA and the designed antisense chimeric RNA. In this model, the sense genomic strands of JAZF1 and SUZ12 genes form an imperfect DNA stem. The RNA/DNA hybrid formed between antisense chimeric RNA (in light and dark blue) and genomic DNA (in black) is indicated by shaded region. The chimeric RNAs are designed with 50 nts targeting JAZF1 gene and 50 nts targeting SUZ12 gene. See supplementary information for chimeric RNA designs. **(C)** RT-PCR detection of induced JAZF1-SUZ12 fusion transcript. Top panel: Nested primers designed to cross the JAZF1-SUZ12 fusion RNA junction. The JAZF1-SUZ12 fusion RNA found in endometrial stromal sarcoma has an RNA junction consist of JAZF1 exon 3 spliced to SUZ12 exon 2. Lower panels: RT-PCR detection of JAZF1-SUZ12 fusion transcript induced by chimeric RNAs in hESC cells. Thirteen out of fifty-one designed antisense chimeric RNAs led to clear induction of JAZF1-SUZ12 fusion transcripts amplified in a double-band pattern (asJS-1, -2, -8, -11, -14, -31, -36, -38, -39, -44, -45, -51, -53). Notation: the prefix ‘as’ stands for ‘antisense’, ‘JS’ stands for ‘JAZF1-SUZ12’, ‘M’ as DNA markers. As controls, transfection with a parental plasmid expressing mCherry (lane 52), cells without transfection (lane 53), and PCR reaction without cDNA (lane 54) all resulted in the absence of induced fusion transcripts. **(D**) Sanger sequencing confirmation of the induced JAZF1-SUZ12 fusion transcript. Each of the thirteen positive double bands was sequenced and results revealed that they contain the same JAZF1-SUZ12 RNA fusion junction sequence. An example of the RNA fusion junction sequence between JAZF1 exon-3 and SUZ12 exon-2 is shown. The full-length Sanger sequencing results are shown in S1 and S2 Figs. **(E)** The intron locations targeted by chimeric RNAs for the thirteen positive cases. These effective locations appeared to be scattered without a clustered hot spot. **(F)** RNA-driven gene fusion is both sequence-specific and cell type-specific. Chimeric RNA “asJS-53” designed to target JAZF1 and SUZ12 induced JAZF1-SUZ12 but not TMPRSS2-ERG in hESC cells (lane 3 and 4, upper vs. lower panel). Conversely, chimeric RNA “antisense-5” targeting TMPRSS2 and ERG induced TMPRSS2-ERG but not JAZF1-SUZ12 fusion in LNCaP cells (lane 6, upper vs. lower panel). Furthermore, JAZF1-SUZ12, an endometrial cancer fusion gene was induced in endometrial cells (hESC), but not in prostate cells (LNCaP) (lane 3 and 4 vs. lane 7 and 8). Conversely, TMPRSS2-ERG, a prostate cancer fusion gene, was induced in prostate cells, but not in endometrial cells (lane 6 vs. 2).

One of the most prominent genetic alterations in cancer is gene fusion resulting from chromosomal rearrangements [1]. Fusion genes are important for understanding cancer mechanisms and developing useful clinical biomarkers and anti-cancer therapies [2]. Identifying the cellular mechanisms that give rise to gene fusion would provide important insight in early disease mechanism, and potentially guide design strategies to inhibit the development of future tumors. Fusion gene formation as a result of chromosomal rearrangements is presumed to occur prior to fusion RNA expression. However, studies of various types of cancer have reported the presence of fusion RNA in individuals without detectable fusion genes at the genomic DNA level [3–5]. The observation, that fusion RNA could be present prior to fusion gene formation, raises the provocative hypothesis that fusion RNA, or any cellular RNA with sequence compositions resembling that of fusion RNA, could act as a template to mediate/specify genomic rearrangement by annealing to their parental genes that are in close proximity (Fig 1A) [6–10]. Resolving such an RNA/DNA hybrid by DNA repair mechanisms may yield the final gene fusion. A precedent for this mechanism is found in ciliates [11] where RNA transcribed from the DNA of previous ciliates generation is used as templates to ‘recombine’ DNA segments in the next generation after mating. Rowley and Blumenthal coined this as “the cart before the horse” hypothesis [12], in that “RNA before DNA” defies the conventional order of the central dogma of biology: DNA ➔ RNA➔ protein [13]. Despite the fundamental implications in cancer and mammalian genome stability, RNA-driven genomic rearrangements in mammalian cells remains controversial as it has been difficult to confirm.

In our previous report [14], we experimentally demonstrated that the expression of a short designer RNA with a chimeric sequence resembling that of TMPRSS2 and ERG genes leads to specified TMPRSS2-ERG gene fusion at the DNA level in prostate cell lines (PNT1A and LNCaP cells). The process is specified by the sequence of chimeric RNA involved, and facilitated by DHT (dihydrotestosterone), a testosterone hormone analog. Surprisingly, it is the ‘antisense’ chimeric RNA that is complementary to TMPRSS2 and ERG gene sequences, rather than the ‘sense’ chimeric RNA that resembles TMPRSS2 and ERG gene sequences, that effectively drives TMPRSS2-ERG gene fusion in prostate cells. Such induced gene fusion events are of low frequency. About 1 in 10^4^ to 1 in 10^5^ cells that expressed chimeric RNAs in our experiments were estimated to carry the induced gene fusion [14]. Nonetheless, as postulated by the accepted framework for understanding cancer progression, if a fusion gene (such as TMPRSS2-ERG) provides a survival advantage, a few affected cells in a normal tissue could be conditioned to proliferate abnormally and significantly contribute to the possibility of cancer formation [15,16].

While these striking results support the provocative model that specific RNA can drive the formation of gene fusions in mammalian cells, one fundamental question yet to be answered is whether such an RNA-driven mechanism is generalizable, or rather, a special case unique to prostate cells. In this report, we provide the important evidence that RNA-driven gene fusion is not a mechanism restricted to prostate cells, but permissible to another mammalian cell type when proper requirements are met. This principle is demonstrated using JAZF1-SUZ12 fusion gene in human endometrial stromal cells (hESC), which represents a particularly important experimental system as it is from this model that “the cart before the horse” hypothesis was originally proposed [10,12]. The results of our study demonstrated a second independent case of RNA-driven genomic recombination in mammalian cells and could have fundamental implications in understanding the role of RNA in genome stability.

## Results

### Exogenously expressed chimeric RNAs induce the JAZF1-SUZ12 fusion in endometrial cells

Previously we demonstrated that the expression of short chimeric RNA can induce TMPRSS2-ERG gene fusion in human prostate cells in a sequence dependent manner [14]. To determine whether the same mechanism is generalizable to other mammalian cell types, we chose normal human endometrial stromal cell line (hESC) and JAZF1-SUZ12 fusion gene as our model. JAZF1-SUZ12 (also known as JAZF1-JJAZ1) is a cancer fusion gene found in about 50% of endometrial stromal sarcomas patients [17–19]. JAZF1 is located on chromosome 7 (p15.1), while SUZ12 on chromosome 17 (q11.2) (Fig 1B). Inter-chromosomal fusion between these two genes resulted in the expression of JAZF1-SUZ12 fusion RNA consisting of first three exons of JAZF1 (exons 1–3) and the last 15 exons of SUZ12 (exons 2–16) [19] (Fig 1C). To recapitulate JAZF1-SUZ12 fusion gene formation, we used a hESC cell line that lacks the JAZF1-SUZ12 fusion [20] as our host cell. The absence of JAZF1-SUZ12 fusion RNA expression in this cell line was confirmed experimentally by RT-PCR (Fig 1C, lane 52–53). We transiently transfected hESC cells with plasmid that expresses the custom-designed short chimeric RNA for 3 days. If the expression of the short chimeric RNA leads to a JAZF1-SUZ12 gene fusion, it is expected that the endogenous full-length fusion RNAs would be transcribed from the newly induced JAZF1-SUZ12 fusion gene. Specific nested RT-PCR primers were designed to amplify induced fusion RNAs (Fig 1C and S1 Text).

The chimeric RNA sequences were designed according to our previous publication [14]. Briefly, we used BLAST alignment to identify intron locations where the sense genomic JAZF1 sequence can pair with the sense genomic SUZ12 sequence to form a complementary genomic DNA stem (see Fig 1B, and S2, S3 and S4 Texts). Matching antisense chimeric RNAs were then designed to target the DNA sequences of SUZ12 and JAZF1 introns near the potential genomic DNA stem, so that the resulting RNA/DNA hybrid together with the genomic DNA stem resemble a three-way junction structure that would be transiently stable (Fig 1B, lower panel). Fifty-one such antisense chimeric RNAs were designed and tested in hESC cells. As shown in Fig 1C, thirteen out of fifty-one (about 25%) designed antisense chimeric RNAs were able to induce JAZF1-SUZ12 fusion transcripts in hESC cells manifested in a double-band pattern. (Fig 1C lower panel, asJS-1, -2, -8, -11, -14, -31, -36, -38, -39, -44, -45, -51, -53). Sanger sequencing of the induced double bands from each of the thirteen positive cases revealed that each contains the same JAZF1-SUZ12 fusion RNA junction sequence with JAZF1 exon-3 joined to SUZ12 exons-2 by an annotated splice site (Fig 1D, full-length sequencing results shown in S1 and S2 Figs), which would be expected of mature endogenous fusion mRNA derived from the JAZF1-SUZ12 fusion gene. Sanger sequencing also revealed that the double bands are merely the results of nested primers employed, as the upper band being the amplicon of primer pair 3F2/3R2 and the lower band the amplicon of primer pair 3F3/3R2 (Figs 1C and S1 and S2). Fig 1E shows the intron locations targeted by designed chimeric RNAs for the thirteen positive cases, and these locations appeared to be scattered without a clustered hot spot.

It is important to point out that the induced JAZF1-SUZ12 fusion transcripts, which contain only annotated exon sequences, cannot arise from the sequence of the expression plasmids. This is because that the chimeric RNA sequences encoded in the plasmids are designed to target the introns (Fig 1E) and contain no exon sequence. Second, the precise annotated splice junctions that join the exons of induced fusion transcripts (including the RNA junction that joins JAZF1 exon-3 to SUZ12 exon-2) strongly indicate that they are generated and processed through cellular splicing mechanisms; therefore, the induced fusion transcript is not the result of RT-PCR artifacts produced by template switching. Furthermore, transfection with a parental plasmid expressing mCherry sequence (Fig 1C, lane 52), cells without transfection (Fig 1C, lane 53), and PCR reaction without cDNA served as RT-PCR controls (Fig 1C, lane 54) all resulted in the absence of induced fusion transcripts. In addition, the experiment was repeated independently at least twice starting from cell transfection to RT-PCR, and the results were identical; that is, it is always the same thirteen antisense chimeric RNAs (out of fifty-one designed) that induced fusion transcripts. Together, the data suggest that expression of a chimeric RNA with sequence complimentary to JAZF1 and SUZ12 gene can lead to the induction of a specified JAZF1-SUZ12 fusion in hESC cells.

To test whether the designed chimeric RNAs specify a pair of genes to undergo fusion in a sequence-specific manner, and whether induced gene fusion is cell type-specific, we cross-compared the effect of a chimeric RNA known to induce TMPRSS2–ERG fusion (antisense-5) [14] to that of a chimeric RNA designed to induce JAZF1-SUZ12 fusion (asJS-53) in both prostate and endometrial cells. As shown in Fig 1F, chimeric RNA (asJS-53) designed to target JAZF1 and SUZ12 induced JAZF1-SUZ12 but not TMPRSS2–ERG in hESC cells (Fig 1F, lane 3 and 4, upper vs. lower panel). Conversely, chimeric RNA (antisense-5) targeting TMPRSS2 and ERG induced TMPRSS2–ERG but not JAZF1-SUZ12 fusion in LNCaP cells (Fig 1F, lane 6, upper vs. lower panel). The result indicates that fusion formation is specified by the sequence of chimeric RNA and not the results of secondary effects such as global genomic instability. Furthermore, specific fusion occurs only in the appropriate cell types, that is, JAZF1-SUZ12, an endometrial cancer fusion gene, can be induced in endometrial cells (hESC), but not in prostate cells (LNCaP) (Fig 1F, lane 3 and 4 vs. lane 7 and 8). Conversely, TMPRSS2–ERG, a prostate cancer fusion gene, can be induced in prostate cells, but not in endometrial cells (Fig 1F, lane 6 vs. 2).

### Antisense chimeric RNAs but not their corresponding sense chimeric RNAs effectively induce JAZF1-SUZ12 fusion

**Fig 2:**
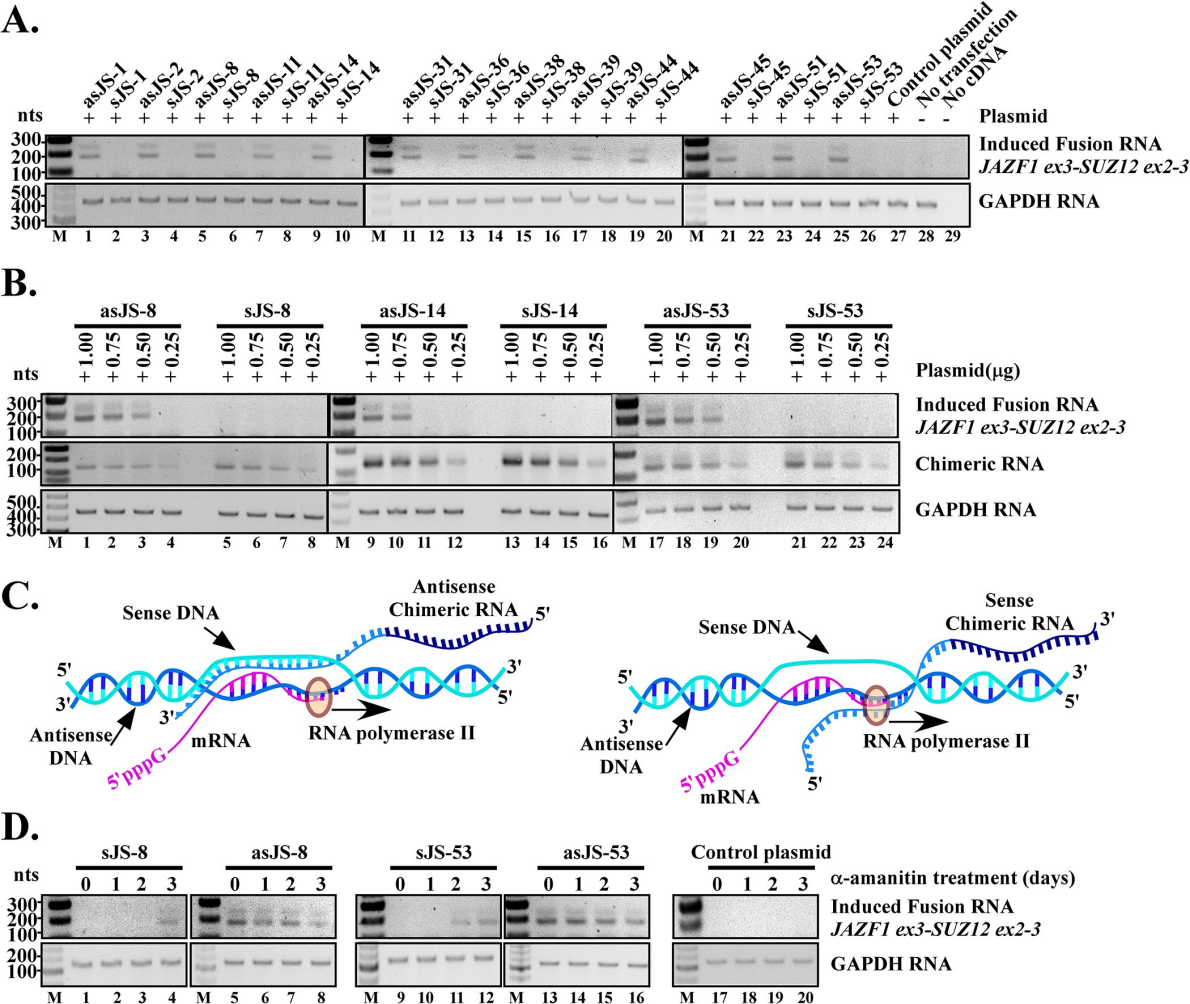
Antisense chimeric RNAs but not their corresponding sense chimeric RNAs induce endogenous JAZF1-SUZ12 fusion transcripts. (**A)** The thirteen positives antisense RNAs (prefix ‘as’) along with their corresponding sense chimeric RNAs (prefix ‘s’) were tested in parallel in hESC cells. RT-PCR results showed that all antisense were able to induce JAZF1-SUZ12 fusion transcript. In contrast, all corresponding sense chimeric RNAs failed to induce fusion transcripts (top panel). The experiment was repeated twice and results were identical. GAPDH serves as internal loading control (bottom panel). **(B)** The inability of sense chimeric RNAs to induce fusion transcripts is not due to the expression levels. The hESC cells were transfected with different amounts of plasmids (1.00μg, 0.75μg, 0.50μg and 0.25μg) expressing either sense or antisense chimeric RNA (middle panel). To maintain the same transfection protocol, mCherry plasmid was added to each transfection to make the final amount of plasmid to 1.0 μg. Experiment was performed for three antisense/sense pairs: asJS-8/sJS-8, asJS-14/sJS-14 and asJS-53/sJS-53. RT-PCR results showed that even when the sense chimeric RNA was intentionally expressed at a much higher level than the antisense chimeric RNA (middle panel, lane 5 vs. 3, 13 vs. 10, and 21 vs. 19), they failed to induce fusion transcripts (top panel, lane 5, 13 and 21). GAPDH serves as internal loading control (bottom panel). **(C)** A model that explains the disparity between antisense and sense chimeric RNA as the result of transcriptional conflict. Left panel: The antisense chimeric RNAs are able to form transiently stable DNA/RNA hybrids with sense strands of genomic DNA. Right panel: In contrast, the sense chimeric RNAs forming DNA/RNA hybrids with antisense strands of genomic DNA (the template strand used for transcription) are likely be “bumped” off by RNA polymerase and unable to stabilize the structures required for initiating genomic rearrangements. **(D)** RT-PCR results that support the transcriptional conflict model. The chimeric RNAs were expressed by U6 (a pol-III promoter) while α-amanitin was used to inhibit pol-II transcription of the parental genes for various time periods (0, 1, 2, and 3 days). α-amanitin was then rinsed off so that the newly induced fusion gene can express the fusion RNA. The induced fusion RNA was then assayed by RT-PCR at day 3. The sense chimeric RNAs that previously failed to induce fusion began to induce JAZF1-SUZ12 (lane 4, 11 and 12) after 3 days of α-amanitin treatment. GAPDH is used as internal loading control.

One important observation from our previous study using TMPRSS2-ERG fusion in prostate cell as the model is that it is the antisense, rather than sense chimeric RNAs, that effectively drive gene fusion [14]. This disparity between antisense and sense occurred even though the sense chimeric RNAs could, in theory, hybridize to the same genomic sites targeted by their antisense counterparts and form similar DNA/RNA hybrids. To examine whether this observation also holds true in the JAZF1-SUZ12 model, a set of sense chimeric RNAs corresponding to the thirteen positive antisense chimeric RNAs were designed and tested in parallel. As shown in Fig 2A, all thirteen antisense chimeric RNAs (prefix”as”, asJS-1 to asJS-53) induced JAZF1-SUZ12 fusion transcript in hESC cells as expected. In contrast, all corresponding sense chimeric RNAs (prefix “s”, sJS-1 to sJS-53) failed to induce JAZF1-SUZ12 fusion transcripts. To test whether the inability of sense chimeric RNAs to induce fusion is due to their expression levels, we selected three pairs of antisense and their corresponding sense chimeric RNAs and expressed them at various levels by transfecting different amount of plasmids. As shown in Fig 2B, sense chimeric RNAs (sJS-8, sJS-14, and sJS-53) failed to induce fusion transcripts even when they were intentionally expressed at a much higher level than the antisense chimeric RNAs (Fig 2B, lane 3 vs. 5, 10 vs. 13, 19 vs. 21). In contrast, the antisense chimeric RNAs require only low levels of expression to induce fusion (Fig 2B, lane 3, 10, and 19). Therefore, consistent with our previous observation [14], it is not the amount of the expressed chimeric RNA but the orientation of the chimeric RNA (antisense vs. sense) that is important for fusion induction. Furthermore, the inability of transfected sense plasmids to induce fusion transcripts also argues against the possibility that plasmid DNA sequences are the source of induced fusion transcripts, as the plasmids expressing sense chimeric RNA contain the same DNA sequences as the plasmids expressing antisense chimeric RNA except that the promoter is placed in the opposite direction (see S3 Fig).

### The antisense versus sense disparity is due to transcriptional activity of parental genes

One plausible explanation for this pronounced disparity between sense and antisense chimeric RNAs in fusion induction is “transcriptional conflict”. It is known that both the JAZF1 and SUZ12 gene are actively transcribed in hESC cells [20]. During transcription, the polymerase-II uses antisense strands of genomic DNA as the template. It would frequently “bump” off sense chimeric RNAs that form DNA/RNA hybrids with the antisense strands of JAZF1 genomic DNA, therefore unable to stabilize the transient structures required for initiating genomic rearrangements (See illustration in Fig 2C). If this hypothesis is correct, shutting down polymerase-II activity should diminish the disparity between antisense versus sense chimeric RNAs. To test this idea, we inhibited the endogenous JAZF1 and SUZ12 gene transcription by α-amanitin, an antagonist specific to RNA polymerase-II [21,22], while continuously expressing the chimeric RNAs using U6 promoter, a polymerase-III promoter insensitive to α-amanitin. This maneuver minimizes transcriptional conflict, and allows the comparison of the effectiveness of sense vs. antisense chimeric RNA on a relatively equal footing. As shown in Fig 2D, the sense chimeric RNAs that previously failed to induce JAZF1-SUZ12 (lane 1 and 9) began to induce after 2 to 3 days of α-amanitin treatment (lane 4, 11, and 12). This latent induction exhibited by sense chimeric RNAs is not a property of unspecific toxicity of α-amanitin because the subtle toxicity of α-amanitin clearly showed the opposite effect on fusion induction. As revealed in Fig 2D, α-amanitin treatment reduced, rather than increased, the fusion induction by antisense chimeric RNA (compare lane 5 vs. 8, and lane 13 vs. 16), presumably because that gene fusion involves complex machinery and requires healthy cellular conditions. An additional control using a parental plasmid vector lacking the sense chimeric RNA sequences failed to induce fusion under the same α-amanitin treatment, indicating again that the latent induction by sense chimeric RNAs is not a property of unspecific toxicity of α-amanitin. The results suggest that shutting down transcription effectively diminishes the antisense versus sense disparity and allows the sense chimeric RNAs to induce gene fusion.

### Hormones inhibit chimeric RNA-induced JAZF1-SUZ12 fusion in hESC cells

**Fig 3:**
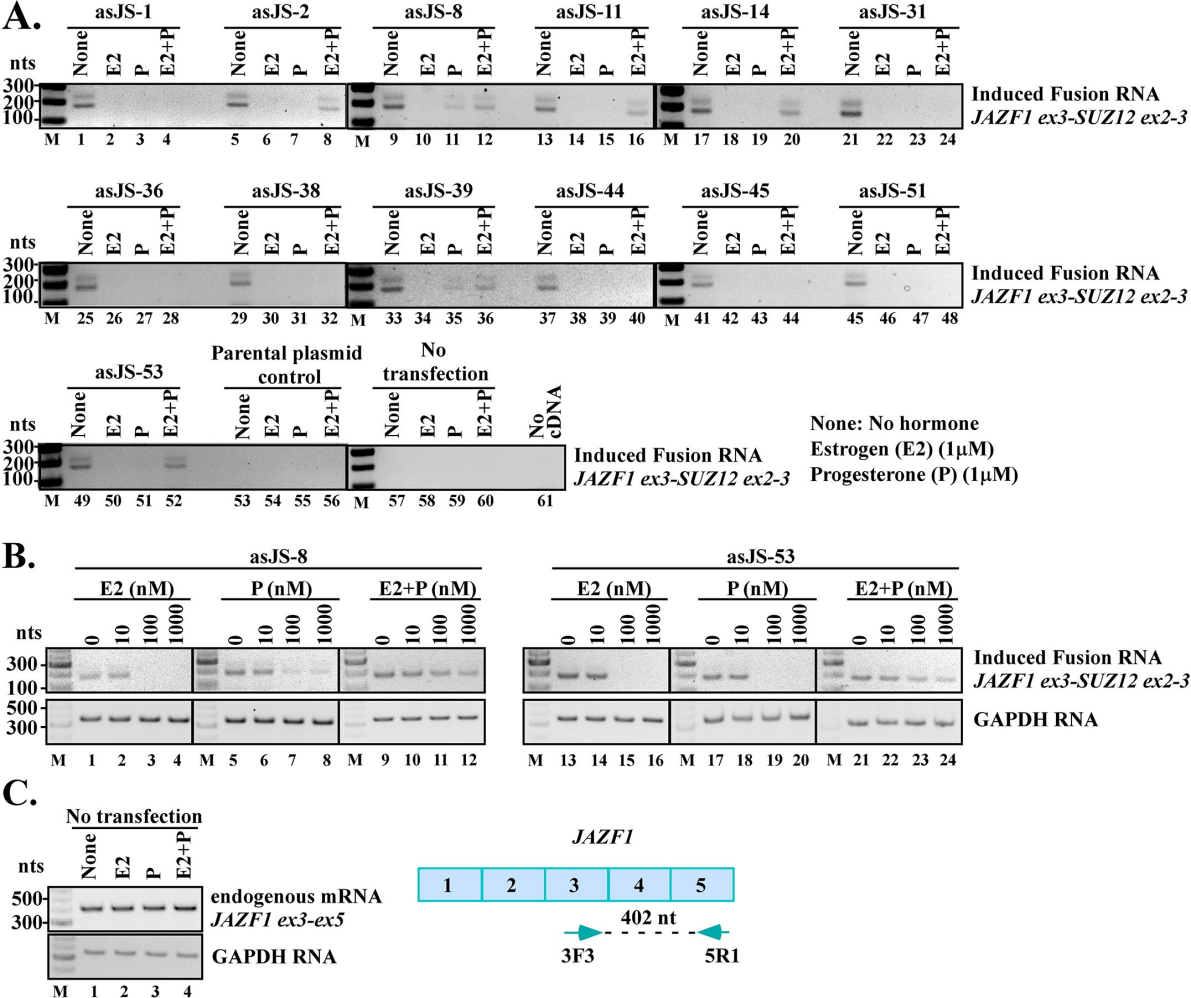
Effects of estrogen and progesterone on JAZF1-SUZ12 fusion induction. **(A)** Antisense chimeric RNAs were expressed in hESC cells followed by treatment with either estrogen (E2, 1μM) or progesterone (P, 1μM) or both (E2 1μM + P 1μM) for three days. RT-PCR results indicate that treatment with estrogen or progesterone consistently inhibited JAZF1-SUZ12 fusion induction in all the thirteen cases of antisense chimeric RNAs tested (None vs. E2 or P). However, the inhibitory effect was lessened in some cases when both hormones were combined (E2+P vs. E2 or P). **(B)** Hormone dosage effects on JAZF1-SUZ12 induction. RT-PCR results indicate that treatment with estrogen or progesterone at 10nM had no effect on JAZF1-SUZ12 induction. More effective hormone concentrations at 100nM or 1μM consistently inhibited the JAZF1-SUZ12 fusion transcripts induced by antisense chimeric RNAs tested (asJS-8 and asJS-53). **(C)** In the absence of antisense chimeric RNAs, estrogen or progesterone has little or no effect on parental JAZF1 gene activity in hESC cells (lane 1 vs, 2, 3, and 4), suggesting that the activity of JAZF1 promoter is not inhibited by estrogen or progesterone. Since the parental JAZF1 gene and the JAZF1-SUZ12 fusion gene have the same promoter, the reduced fusion transcript suggests that it is the process of JAZF1-SUZ12 fusion gene formation that is suppressed by estrogen or progesterone. The primers used to detect the JAZF1 mRNA are shown on the right. Notation: ‘E2’ for estrogen, ‘P’ for progesterone, ‘None’ for no treatment.

Previously we have shown that chimeric RNA-induced TMPRSS2-ERG fusion in prostate cells requires testosterone [14], a male hormone known to alter chromosomal DNA looping therefore the physical proximity between TMPRSS2 and ERG genes that may facilitate gene fusion [23–25]. The female hormone estrogen and progesterone are also known to alter chromosomal DNA looping [26] and play a role in DNA damage [27]. To examine the effects of female hormones on JAZF1-SUZ12 fusion formation, we tested the antisense chimeric RNAs in combination with estrogen and/or progesterone, hormones known to influence the physiology of endometrial cells. First, we expressed the antisense chimeric RNAs in hESC cells by transfection. This was then followed by three days of treatment with 1μM estrogen or 1μM progesterone or both. As shown in Fig 3A, treatment with either estrogen (E2) or progesterone (P) did not increase the induction of JAZF1-SUZ12 fusion transcripts; rather, they inhibited it (Fig 3A, None vs. E2 or P). This inhibitory effect was consistently observed in all thirteen cases of antisense chimeric RNAs tested. To further examine the hormonal effects at different concentrations, we focused on two antisense chimeric RNAs (asJS-8 and asJS-53). The effective hormone concentrations in hESC cells were measured by the responses of well-established target genes (FOXO1A to progesterone and PR to estrogen) with hormones at 0nM, 10nM, 100nM and 1μM [28]. As shown in S4 Fig, the hESC cells responded to estrogen and progesterone moderately at 10nM and more robustly at 100nM or 1μM. Consistent with this range of effective concentrations, hormones at 10nM showed no inhibitory effect on fusion induction in hESC cells, whereas hormone concentrations at 100nM or 1μM clearly inhibited the induction of JAZF1-SUZ12 (Fig 3B). Furthermore, treating the hESC cells with estrogen or progesterone at 1μM (the highest concentration used in this study) had little or no effect on parental JAZF1 gene expression (Fig 3C, None vs. E2 or P), indicating that neither estrogen nor progesterone inhibits JAZF1 promoter activity. Therefore, the observed reduction of JAZF1-SUZ12 fusion expression is not due to hormonal inhibition of JAZF1 promoter that now drives the JAZF1-SUZ12 fusion gene. This implies that the aberrant JAZF1-SUZ12 gene fusion process is suppressed by estrogen or progesterone. Intriguingly, in many cases, the inhibitory effect of individual hormones was lessened when estrogen and progesterone are combined (E2 + P, lane 8, 12, 16, 20, 36, 52), which may suggest that DNA looping is altered differently in the presence of both hormones as compared to individual hormones.

### Induced JAZF1-SUZ12 fusion is the result of genomic rearrangements

**Fig 4:**
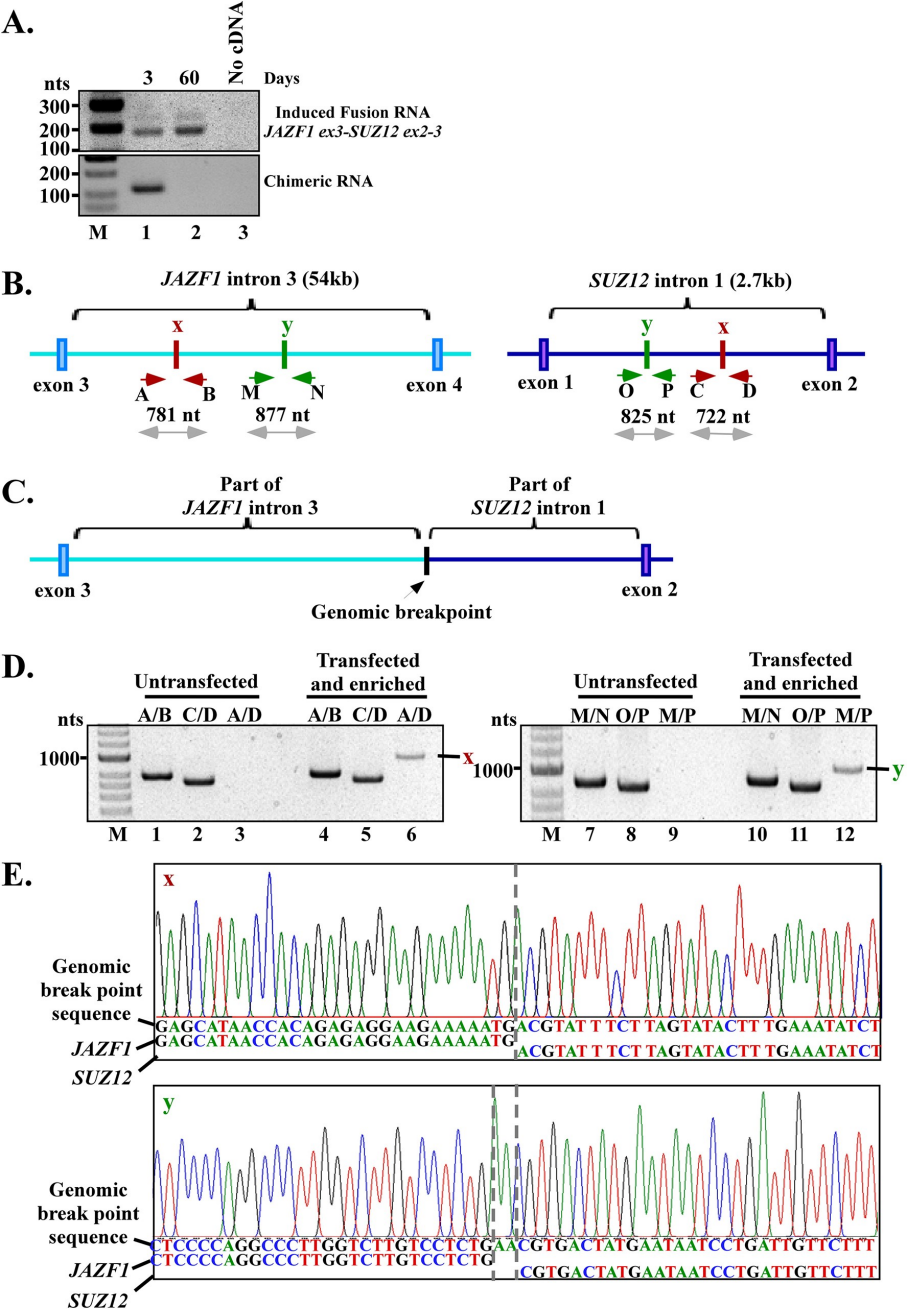
Induced JAZF1-SUZ12 fusion is the result of genomic rearrangements. **(A)** RT-PCR shows the transient nature of exogenously expressed chimeric RNA that was degraded and completely absent by day 60 (lower panel, lane 1 vs. 2), and the persistent nature of the induced fusion transcript (upper panel, lane 1 vs. 2) which was continuously expressed up to day 60 in the enriched cell population. This shows that the continuously expressed JAZF1-SUZ12 fusion RNA does not require the presence of chimeric RNAs. See S5 Fig for procedures used to propagate and enrich the induced hESC population. **(B)** The wild-type alleles with two identified genomic breakpoints marked as ‘x’ and ‘y’, and the primers used to amplify these breakpoints. The intron sizes are not presented in proportion as JAZF1 intron-3 (54kb) is much larger than SUZ12 intron-1 (2.7kb). **(C)** Schematics of the rearranged allele of the final fusion gene with JAZF1 and SUZ12 joined at the intron breakpoints. **(D)** The unrearranged wild-type JAZF1 allele near breakpoint ‘x’ was amplified by primer pair A/B (781 bp; lanes 1 and 4) and near breakpoint ‘y’ by primer M/N (877 bp; lanes 7 and 10). The unrearranged wild-type SUZ12 allele near breakpoint ‘x’ was amplified by primer pair C/D (722 bp; lanes 2 and 5) and near breakpoint ‘y’ by primer O/P (825 bp; lanes 8, and 11). The genomic fusion band ‘x’ (951 bp) revealed by fusion-specific primer pair A/D, and fusion band ‘y’ (976 bp) revealed by primer pair M/P, were present only in the enriched hESC population but absent in untransfected hESC cells (lane 6 vs. 3, and lane 12 vs. 9). See S6 Fig for multiplex primer designs used for initial scanning of potential breakpoints. **(E)** Sanger sequencing of the ‘x’ and ‘y’ fusion band identified the exact genomic breakpoints marked by dash lines. The genomic breakpoint ‘y’ contains a ‘AA’ insertion (marked by dash lines). The full-length Sanger sequences of 951 bp for “x” and 976 bp for “y” are shown in S7 and S8 Figs.

To investigate whether the induced JAZF1-SUZ12 fusion transcript is the result of JAZF1-SUZ12 gene fusion at the DNA level via genomic rearrangement, we continued to propagate and enrich the transfected hESC population by dividing and testing the subpopulations for JAZF1-SUZ12 fusion transcript continuously for 60 days (Procedure used to propagate and enrich the induced hESC population is shown in S5 Fig). As shown in the lower panel of Fig 4A, antisense chimeric RNA (asJS53) transiently expressed by plasmids was degraded and undetectable by day 60, indicating its transient nature (lane 1 vs. 2, lower panel). In contrast, the induced JAZF1-SUZ12 fusion transcript was continuously expressed at day 60 in the absence of antisense chimeric RNA (lane 1 vs. 2, upper panel), indicating the permanent nature of the induced fusion transcript. This result indicates that the sustained expression of induced JAZF1-SUZ12 fusion transcripts does not require the continuous presence of chimeric RNA, therefore suggesting that the induced JAZF1-SUZ12 fusion transcript is the consequence of gene fusion at the DNA level.

The definite proof of gene fusion via genomic rearrangement was provided by genomic PCR that identifies the genomic DNA breakpoints induced by antisense chimeric RNA. First, we harvested the genomic DNA from the transfected and enriched hESC population as well as from untransfected hESC cells that served as the negative control. We then designed a series of specific primer pairs for mapping the genomic breakpoints (Fig 4B, and multiplex primer designs shown in S6 Fig). The results of multiplex genomic DNA PCR identified two distinct genomic breakpoints (labeled as “x” and “y”) between the JAZF1 and SUZ12 genes (Fig 4B). Based on these breakpoints, the rearranged allele of the final fusion gene with JAZF1 and SUZ12 joined at the intron breakpoints is shown as a schematics in Fig 4C. Both the genomic breakpoint “x” and “y” were present only in the transfected/enriched hESC population but absent in untransfected hESC cells. As shown in Fig 4D, for the breakpoint “x”, the unrearranged wild-type JAZF1 and SUZ12 alleles were amplified by gene-specific primer pair A/B and C/D respectively, in untransfected cells (Fig 4D, lane 1 and 2) and transfected/enriched hESC population (Fig 4D, lane 4 and 5), indicating that both populations contain intact alleles. In contrast, a genomic DNA fusion band “x” of 951 bp amplified by fusion-specific primer pair A/D was present only in the transfected/enriched hESC population (lane 6) and absent in untransfected hESC cells (lane 3), indicating that the genomic DNA fusion band “x” was the result of antisense chimeric RNA expression. Similarly, for the breakpoint “y”, the unrearranged wild-type JAZF1 and SUZ12 alleles were amplified by gene-specific primer pair M/N and O/P respectively in both untransfected cells (Fig 4D, lane 7 and 8) and transfected/enriched hESC population (Fig 4D, lane 10 and 11). In contrast, a genomic fusion band “y” of 976 bp amplified by fusion-specific primer pair M/P was present only in the transfected/enriched hESC population (lane 12) and absent in untransfected hESC cells (lane 9). These results confirm that there were no DNA rearrangements at these specific alleles in parental hESC cells, and that the genomic DNA rearrangements at these alleles were induced by antisense chimeric RNAs.

Sanger sequencing of the excised DNA fusion bands (Fig 4D, lane 6 and 12) revealed the exact genome coordinates at single base resolution for breakpoint “x” and “y”. Both “x” and “y” are located in JAZF1 intron-3 (“x”- chr7:27877982, “y”- chr7:27870597, GRCh38/hg38) and in SUZ12 intron-1 (“x”- chr17:31938610, “y”- chr17: 31938260, GRCh38/hg38). Fig 4E shows the Sanger sequencing results near the breakpoints. The full-length Sanger sequences of 951 bp for “x” and 976 bp for “y” are shown in S7 and S8 Figs. We observed no obvious sequence homology between JAZF1 and SUZ12 at both genomic breakpoints (Fig 4E), suggesting that this gene fusion is mediated by non-homologous break/repair mechanisms [29,30]. The identified DNA breakpoints indicate that a new intron was formed between JAZF1 and SUZ12 in the fusion gene (Fig 4C). Splicing of pre-mRNA derived from the JAZF1-SUZ12 fusion gene would remove the new intron sequence, resulting in a mature mRNA with an RNA junction of JAZF1 exon-3 joined to SUZ12 exons-2 by the annotated splice site, which was exactly the result we found in Fig 1C and 1D. In other words, the evidence of genomic breakpoints found in JAZF1 intron-3 and in SUZ12 intron-1 is consistent with the observed JAZF1-SUZ12 fusion RNA junction found at the RNA level. These results strongly support that the induced JAZF1-SUZ12 is the consequence of gene fusion at the DNA level caused by genomic rearrangements.

## Discussion

Earlier we reported an unappreciated RNA-driven mechanism in which the expression of a designer chimeric RNA induces specified TMPRSS2-ERG gene fusions in human prostate cells. One fundamental question is whether such RNA-driven gene fusion mechanism is generalizable, or a unique case restricted to prostate cells. In this report, we provide compelling evidence that expression of a designer chimeric RNA targeting JAZF1 and SUZ12 gene drives the formation of JAZF1-SUZ12 gene fusion in human endometrial cells. Our study utilizes JAZF1 and SUZ12 genes that are unrelated to TMPRSS2 and ERG genes, and the gene fusion is induced in a human endometrial cell line, an entirely different cell type from the prostate cell. Therefore, the study represents an independent exemplar of RNA-driven gene fusion in mammalian cells. The experimental outcomes as we have documented here demonstrated that the mechanism is not restricted to prostate cells but readily extended to endometrial cells. The results therefore represent an important stepping-stone in establishing the generality of RNA-driven genomic recombination in mammalian cells.

The rules that govern the RNA-driven JAZF1-SUZ12 gene fusion in endometrial cells, as demonstrated in this report, appear remarkably similar to that of RNA-driven TMPRSS2-ERG fusion in prostate cells. First, the chimeric RNAs designed with 50 nt complementary to JAZF1 introns and 50 nt complementary to SUZ12 introns were able to induce JAZF1-SUZ12 fusion in hESC cell. The results confirm that this length of chimeric RNA used to target introns, a design principal derived from previous TMPRSS2-ERG fusion study [14], is sufficient to engage a parental gene for fusion event in mammalian cells. Yet not all designer chimeric RNAs are effective, about 1 in 4 (13 out of 51) designed chimeric RNAs in our study were effective in driving JAZF1-SUZ12 gene fusion (Fig 1C). This percentage is similar to that of the chimeric RNAs designed to target TMPRSS2-ERG fusion reported previously. The data suggest that other factors that are yet to be identified, such as the genomic DNA sequence composition required for targeting, and whether the target sites are accessible for forming RNA/DNA hybrids, could play roles in determining fusion induction efficiency. Future studies focusing on these parameters could provide important insights in developing chimeric RNA-based gene-editing technology via mechanisms native to mammalian cells.

The second strong mechanistic similarity between RNA-driven JAZF1-SUZ12 gene fusion in endometrial cells and RNA-driven TMPRSS2-ERG fusion in prostate cells is that the antisense rather than sense chimeric RNAs effectively drive gene fusion. Of thirteen antisense chimeric RNAs that were demonstrated to be capable of inducing JAZF1-SUZ12 fusion, all the corresponding sense chimeric RNAs failed to induce fusion (Fig 2A). This was true even when the sense chimeric RNA was deliberately expressed at a much higher level than the antisense chimeric RNA (Fig 2B). The same ‘sense vs. antisense’ disparity was also pronounced in chimeric RNA-induced TMPRSS2-ERG fusion in prostate cells [14]. In both cases, α-amanitin treatment, which shuts down polymerase-II mediated transcription, effectively diminished this disparity and enabled the sense chimeric RNAs to induce gene fusion (Fig 2D) [14]. These data, derived from two independent cases, support a model where the chimeric RNA sequence invades the target gene DNA sequence to stabilize a transient RNA/DNA duplex reminiscent of an R-loop (Fig 1A) [31–35]. Because the JAZF1 and SUZ12 promoters are highly active in hESC cells [20], sense chimeric RNAs forming DNA/RNA hybrids with antisense strands of genomic DNA (the template strand used for transcription) would be frequently “bumped” off by polymerase-II (Fig 2C, right panel). In contrast, antisense chimeric RNA, which hybridizes to the sense strands of genomic DNA (the non-template strand), would have increased probability to stabilize the transient DNA/RNA hybrid required for initiating genomic rearrangements (Fig 2C, left panel). The disparity suggests that when the parental genes are active, the antisense chimeric RNA or a cellular RNA with sequence resembling an antisense chimeric RNA, could be ‘the cart before the horse’ that effectively drives gene fusion. However, our results also showed that α-amanitin treatment enables the sense chimeric RNAs to induce gene fusion. Therefore, under cellular conditions such as when the parental gene transcriptions are inactive, sense chimeric RNAs, or any cellular RNA with sequence resembling a sense chimeric RNA [5,7,36], could be effective in driving genomic rearrangements. Nevertheless, when the parental genes are inactive, the resulting fusion genes will also be inactive, and neither fusion RNAs nor fusion proteins will be produced from the induced fusion gene. Such ‘silent’ fusion genes likely bear little biological consequences, and their presence would be difficult to confirm as they can’t be detected at the RNA or protein level.

One immediate question that arises from the ‘sense vs. antisense’ disparity is whether such an antisense chimeric RNA capable of acting as the ‘initiator’ to induce JAZF1-SUZ12 fusion does exist and where it might originate? Previously in our study of TMPRSS2-ERG gene fusion, we identified a cellular AZI1 RNA with sequence ‘partially’ complementary to TMPRSS2 and ERG genes [14]. When AZI1 RNA, but not its protein, was expressed in prostate cells, it induced gene fusion between TMPRSS2 and ERG. The discovery that the endogenous cellular AZI1 RNA can act as an ‘initiator’ chimeric RNA to induce TMPRSS2-ERG fusion, indicates that ‘antisense-like’ RNAs do not have to arise from the parental genes that are targeted for fusion, but arise from unrelated genomic sources that resemble an imperfect chimeric RNA antisense to both parental genes. However, identifying such ‘antisense-like’ initiator RNAs by bioinformatics is challenging, as RNA-DNA hybrids may involve atypical non-Watson–Crick base pairings such as G-U paring as well as mismatches. Nevertheless, the data in this report show that RNA-driven JAZF1-SUZ12 fusion is mechanistically plausible and that the necessary ‘machinery’ are clearly present in endometrial cells. Moreover, the sequences of effective designer antisense chimeric RNAs identified in this report could provide the sequence composition to search for cellular ‘antisense-like’ RNAs that may initiate JAZF1-SUZ12 gene fusion. If such cellular RNAs with partial sequence complementarity and ‘antisense’ to the JAZF1 and SUZ12 gene could be identified in the future, it could have important biological relevance and provide important insights into the disease origin of JAZF1-SUZ12 fusion in endometrial stromal sarcomas, and potentially lay the foundation for preventive and targeted intervention. Furthermore, with new chimeric RNAs increasingly been identified from various cell lines and tissues including tumors, comprehensive chimeric RNA databases such as ‘ChiTaRS’ [37] could provide valuable sources for searching for chimeric RNAs ‘antisense’ to two parental genes. Testing these candidate chimeric RNAs experimentally could lead to the identification of ‘initiator’ chimeric RNA capable of inducing gene fusion in their appropriate cells thus providing important early disease mechanisms related to the formation of cancer fusion genes.

A third important similarity between RNA-driven JAZF1-SUZ12 gene fusion in endometrial cells and RNA-driven TMPRSS2-ERG fusion in prostate cells is that they both are influenced by hormone, albeit in the opposite directions. Previously we showed that testosterone ‘facilitates’ RNA-driven TMPRSS2-ERG fusion in prostate cells [14]. In contrast, our current study indicates that estrogen or progesterone ‘suppresses’ RNA-driven JAZF1-SUZ12 fusion in endometrial cells. The inhibitory effect of estrogen or progesterone was consistently observed in all antisense chimeric RNAs tested in hESC cells regardless of their targeting sequences (Fig 3A). The data suggests that estrogen and progesterone may possess a general ‘protective effect’ in preventing aberrant JAZF1-SUZ12 fusion formation and that female patients with declined estrogen or progesterone levels are possibly more susceptible to JAZF1-SUZ12 fusion formation. Consistent with this notion, it is worth noting that JAZF1-SUZ12, which is a cancer fusion gene found in about 50% of the patient population with endometrial stromal sarcomas, occurs primarily in premenopausal women between ages of 45 and 50 years when hormone levels are falling [38,39].

The mechanism that underlies the hormonal effect on JAZF1-SUZ12 gene fusion formation in endometrial cells is yet to be understood. Several studies indicated that the close proximity of the parental genes in genome 3D organization may contribute to the occurrence of gene fusion events [40–43]. In prostate cells, it is known that binding of testosterone to androgen receptors and genomic DNA alters chromosomal looping and brings TMPRSS2 gene and ERG gene in close physical proximity that may facilitate TMPRSS2-ERG gene fusion [23–25]. Similarly, estrogen and progesterone have been shown to act as local and global genome 3D organizers through hormone-activated receptors [26,44,45] although their specific DNA looping effects on JAZF1 and SUZ12 gene are unknown. We propose that the effects of estrogen and progesterone on DNA looping may further separate JAZF1 gene and SUZ12 gene apart in genome 3D organization, hence reducing the possibility of JAZF1-SUZ12 gene fusion. Furthermore, it was shown that estrogen and progesterone could have similar or opposite effects on local chromosomal organization depending on the chromosomal regions [26,44,45]. This may explain why the presence of both estrogen and progesterone attenuates the observed inhibition in gene fusion induction from individual hormones (see Fig 3A, E2 + P, lane 8, 12, 16, 20, 36, 52), as local DNA looping could be organized differently in the presence of both estrogen and progesterone as opposed to individual hormones.

In summary, our results demonstrated that transient expression of designer chimeric RNAs in human endometrial stromal cells can recapitulate the formation of JAZF1-SUZ12, a cancer fusion gene commonly found in low-grade endometrial stromal sarcomas. However, it is the antisense rather than sense chimeric RNAs that effectively drive JAZF1-SUZ12 gene fusion. As we have discussed, the rules that govern the RNA-driven JAZF1-SUZ12 gene fusion in endometrial cells share strong similarity to that of RNA-driven TMPRSS2-ERG fusion in prostate cells. Together, these results suggest that RNA-driven genomic rearrangement in different mammalian cells involves similar mechanisms and is generally permissible when proper requirements are met. Although the RNA-driven mechanism may not be the only mechanism that leads to gene fusions, it is the only mechanism shown so far to ‘specify’ the gene partners in a sequence-specific manner to undergo chromosomal rearrangements in mammalian cells. The RNA-driven gene fusion demonstrated in this report could have fundamental implications in the role of RNA in mammalian genome stability [46–48], and provides important insight in early disease mechanisms, as well as developing gene-editing technology via mechanisms native to mammalian cells.

## Materials and methods

### hESC cell culture

hESC cells were routinely cultured in DMEM/ F-12 media (DMEM/ F-12, 50/50 1X, # 10-090-CV, Corning cellgro) containing 10% fetal bovine serum (premium grade FBS, #1500–500, Seradigm), 1% penicillin/streptomycin (#15140–122, Gibco), 1% ITS premix (#354352, Corning) and 1.5g/L Sodium Bicarbonate (# S6014-500G, Sigma) in a 5% CO_2_ humidified incubator.

### Transient transfection of plasmids for expressing the chimeric RNAs

Twenty hours prior to transfection, hESC cells were seeded in 12-wells plate (BioLite 12 Well Multidish, #130185, Thermo Fisher Scientific) with a density of 3.5x10^4^cells/well and 1 ml/well of culture medium. Transfection was performed using 293fectin reagent (#12347–019, Gibco by Thermo Fisher Scientific) according to manufacturer’s protocol. Briefly, 1μg of a particular plasmid was first diluted in 50μl of the serum-free DMEM followed by immediate mixing by pipetting and 3 μl of transfection reagent was diluted in another 50μl of the serum-free DMEM in a separate tube. Both tubes were incubated at room temperature for 5 minutes followed by mixing together in one tube and another round of incubation of 20 minutes. The DNA/transfection reagent mixture was then added drop wise to a well containing hESC cells in 1ml medium.

For enough numbers of cells for RNA preparation and RT-PCR, each experiment was done in 4 wells of 12 well plate.

### Hormones preparation and treatment

Estrogen (E2) (β-Estradiol #E8875-1G) and Progesterone (P) (#P8783-1G) was purchased from Sigma Aldrich. Concentrated stock of 2mM was prepared in 100% ethanol (200 proof ethanol, Koptec, #V1016) and then aliquoted into 1ml per tube and stored at -20°C.

For treating cultured cells, concentrated E2 or P stock was diluted as 10x working solutions (for example, for 1μM final concentration, 10x is prepared as 10 μM) with the appropriate complete culture medium and used immediately. Six hours post transfection, 111μl of fresh 10x E2 or P working solutions was added to each well of 12-wells plate containing 1ml medium and transfected cells.

### RNA isolation

Total RNA from cultured cells was extracted using RiboPure RNA isolation Kit according to manufacturer’s instructions (#AM1924, Invitrogen). Briefly, cells were homogenized in 1000μl of Tri-reagent (provided) and were then lysed with 200μl of chloroform. The sample was then vortexed and incubated at room temperature for 5 min followed by centrifugation at 12,000 x g for 10 min at 4°C to separate the mixture into a lower, red, organic phase, an interphase and a colorless, upper, aqueous phase. Upper aqueous phase was then collected and 200μl of ethanol was added. Each sample was then passed through the filter assembly resulting in the binding of the nucleic acids to the filter. The filter assembly was then rinsed with wash buffer and total RNA then eluted in a new tube for further analysis.

For detection of residual genomic and plasmid DNA, eluted RNA was subject to PCR reaction with primers specific to intron regions of house-keeping gene GAPDH, and with primers specific to plasmid transfected. Total RNA was converted to cDNA only if it is validated as free of DNA contamination.

### Reverse transcription reaction

1 μg of total RNA was used for each reverse transcription reaction according to manufacturer instruction (superscript III RT, # 18080–051, Invitrogen). RNA was converted to cDNA either with Oligo dT primer (for induced fusion transcripts) or with random hexamers (for chimeric RNAs expressed by U6 promoter). After the addition of dNTPs, the mixture was denatured at 65°C for 5 minutes. This was followed by the addition of a master-mix containing 1× superscript buffer, 10 mM DTT, 5 mM Magnesium chloride, RNaseOUT and Superscript III reverse transcriptase. Reactions were carried out at 50°C for 50 minutes and then terminated by incubation at 85°C for 5 minutes. cDNA was then treated with RNase-H for 20 minutes at 37°C to degrade RNA in DNA/RNA hybrid. 5 μl of cDNA was used as template for each subsequent PCR reaction.

### RT-PCR for detecting induced fusion transcripts

PCR for induced fusion RNAs was done with a standard three-step protocol using REDTaq DNA polymerase (#D5684-1KU, Sigma) according to manufacturer instruction.

Reaction was set as follows:

PCR reaction:

    Forward primer: 1.0μl (from 10μM stock, Sigma)

    Reverse primer: 1.0μl (from 10μM stock, Sigma)

    10x reaction buffer: 5.0μl (comes with REDTaq, Sigma)

    dNTPs: 1.0μl (from 10mM stock, #11969064001, Roche)

    DMSO: 1.5μl (#154938, Sigma-Aldrich)

    cDNA: 5.0μl (from 20μl stock prepared from 1 μg RNA)

    Autoclaved Milli-Q water: 35.5μl

REDTaq DNA polymerase: 1.0μl (#D5684-1KU, Sigma)

Total volume: 50μl

PCR conditions for three-round nested PCR for *JAZF1*-*SUZ12*:

    1st round: PCR with JAZF1 ex-3 F1 and SUZ12 ex-3 R1 on 4μl of cDNA

    Pre-denaturation 94°C, 4 min

    Denaturation 94°C, 30 sec

    Annealing 57°C, 45 sec

    Extension 72°C, 60 sec

    Repeat Denaturation-Annealing-Extension cycle 35 times for induced fusion RNA

    Final Extension 72°C, 5 min

    2nd round: PCR with *JAZF1* ex-3 F2 and *SUZ12* ex-3 R2 on 3μl of 1st round product, PCR conditions same as 1st round.

    3rd round: PCR with *JAZF1* ex-3 F3 and *SUZ12* ex-3 R2 on 3μl of 2nd round product, Annealing temperature is 52°C.

### PCR for detecting genomic DNA breakpoint

    For genomic breakpoint mapping, PCRs were done with a standard two-step protocol using REDTaq DNA polymerase (#D5684-1KU, Sigma) according to manufacturer instruction.

    Reaction was set as follows:

PCR reaction:

    Forward primer: 1.0μl (from 10μM stock, Sigma)

    Reverse primer: 1.0μl (from 10μM stock, Sigma)

    10x reaction buffer: 5.0μl (comes with REDTaq, Sigma)

    dNTPs: 1.0μl (from 10mM stock, #11969064001, Roche)

    DMSO: 1.5μl (#154938, Sigma-Aldrich)

    Genomic DNA: 200 ng

    Autoclaved Milli-Q water: to make final volume 50.0 μl

REDTaq DNA polymerase: 1.0μl (#D5684-1KU, Sigma)

Total volume: 50μl

2μl from the above reaction (1st round PCR) was used as template for the 2nd round PCR.

### Cloning and sanger sequencing of induced fusion transcripts

PCR amplified cDNA bands were excised from the gel and eluted using QIAquick Gel Extraction Kit (#28706, Qiagen). The eluted bands were then cloned to pGEM-T vector (pGEM-T vector system I, # A3600) following manufacturer instruction. Sanger sequencing was performed using the service of Eurofins Genomics.

### α-Amanitin assay

Twenty hours prior to transfection, hESC cells were seeded in 12-wells plate (BioLite 12 Well Multidish, #130185, Thermo Fisher Scientific) with a density of 3.5x10^4^cells/well and 1 ml/well of culture medium. Transfection was performed using 293fectin reagent (#12347–019, Gibco by Thermo Fisher Sceintific) as described earlier. Following overnight incubation, cells were then treated with 0.25 μg/ml α-amanitin for various time periods (0, 1, 2 and 3 days). Cells were then revived in fresh medium without α-amanitin for 4 days and RT-PCR was performed for JAZF1-SUZ12 fusion.

## Supporting information

S1 Fig Sanger sequencing confirmation of the upper induced *JAZF1-SUZ12* fusion RNA band amplified by the nested primer pair 3F2-3R2. Induced fusion transcripts were converted to cDNA using oligo dT primers and PCR was performed using nested primers targeting *JAZF1* exon-3 and *SUZ12* exon-3 as described in Fig 1C. As discussed in the main text, the RT-PCT yielded a double-band pattern. The upper band was cut out followed by Sanger sequencing. The result confirms that it was amplified by nested primer pairs 3F2 (light blue arrow in *JAZF1* exon-3) and 3R2 (dark blue arrow in *SUZ12* exon-3). Sanger sequencing chromatograms also confirmed that the induced fusion transcript contains *JAZF1* exon-3, *SUZ12* exons-2 and exons-3, and that these exons were joined by splicing at the annotated splice sites as would be expected of mature endogenous *JAZF1-SUZ12* fusion mRNA. We sequenced each of the thirteen upper bands shown in Fig 1C of the main text, and confirmed that they contain the same JAZF1-SUZ12 fusion RNA sequence amplified by nested primer pairs 3F2 and 3R2. Only the Sanger sequencing result of RT-PCR band induced by asJS-8 is shown here as an example. The pGEM-T plasmid was used for cloning the cDNA fragments.
(TIF)

S2 Fig Sanger sequencing confirmation of the lower induced *JAZF1-SUZ12* fusion RNA band amplified by the nested primer pair 3F3-3R2. Similar to what is described in S1 Fig, the lower RT-PCR band was cut out followed by Sanger sequencing. The result confirms that it was amplified by nested primer pairs 3F3 (light blue arrow in *JAZF1* exon-3) and 3R2 (dark blue arrow in *SUZ12* exon-3). The results confirmed that the lower induced fusion transcript contains *JAZF1* exon-3, *SUZ12* exons-2 and exons-3, and that these exons were joined by splicing at the annotated splice sites as would be expected of mature endogenous *JAZF1-SUZ12* fusion mRNA. We sequenced each of the thirteen lower bands shown in Fig 1C of the main text, and confirmed that they contain the same JAZF1-SUZ12 fusion RNA sequence amplified by nested primer pairs 3F3 and 3R2. Only the Sanger sequencing result of RT-PCR band induced by asJS-8 is shown here as an example. The pGEM-T plasmid was used for cloning the cDNA fragments.
(TIF)

S3 Fig Schematics of the plasmids used to express designed chimeric RNAs in sense or antisense orientation. The chimeric RNAs are designed with 50 nts targeting *JAZF1* gene and 50 nts targeting *SUZ12* gene, and expressed either in the sense or antisense orientation using the U6 promoter. Plasmid expressing sense chimeric RNA contains the same DNA sequences as the plasmid expressing antisense chimeric RNA except that the U6 promoter is placed in the opposite direction. ‘ts’ represents the transcriptional stop sequence “TTTTTT” for U6 promoter.
(TIF)

S4 Fig The effective hormone concentrations in hESC cells measured by the responses of target genes. The hESC cells were treated with different concentrations (0nM, 10nM, 100nM and 1μM) of either estrogen (E2) or progesterone (P) or both (E2 + P) for three days. RT-PCR was performed for FOXO1A (a progesterone target gene, upper panel) and PR (an estrogen target gene, lower panel) respectively. The results indicate that hESC cells responded to estrogen and progesterone moderately at 10nM and more robustly at 100nM or 1μM.
(TIF)

S5 Fig Procedure used to propagate and enrich the induced hESC population. The induced *JAZF1-SUZ12* gene fusions are of low frequency events. To increase the percentage of positive cells in the population, hESC cells were transfected consecutively five times with ‘asJS-8’, and then divided into sub-populations. Half of the cells from each sub-population were harvested for RT-PCR assays to detect the presence of induced fusion transcript, whereas the other half was used for continuous propagation of the population. The sub-population containing the highest intensity of induced fusion transcripts (circled in red) was used for the next round of division, RT-PCR assays, and continuous propagation.
(TIF)

S6 Fig Locations of multiplex primers used for initial mapping of genomic breakpoints. The locations of 56 forward primers (light blue) spacing across *JAZF1* intron-3 (~54 kb) and 10 reverse primers (dark blue) spacing across *SUZ12* intron-2 (~2.7 kb) are shown. Each vertical line represents a target location by a nested primer set. The locations of identified genomic breakpoint ‘x’ and ‘y’ are marked by dark green lines. The specific primers that initially identified the genomic breakpoint ‘x’ and ‘y’ are labeled as red.
(TIF)

S7 Fig Sanger sequencing of the genomic breakpoint ‘x’ confirmed that the induced *JAZF1-SUZ12* fusion is the result of genomic rearrangement. hESC cells transfected with asJS-8 were enriched as described in S5 Fig. Genomic DNA PCR performed on the enriched hESC population yielded a gene fusion band ‘x’ of ~951 bp as shown in Fig 4D of the main text. Sanger sequencing confirmed that the 951 bp fusion DNA band contains ~501 bp of *JAZF1* intron-3 joined to ~450 bp of *SUZ12* intron-1. The locations of the primers used to amplify the fusion DNA product are marked as the light blue and dark blue arrows in *JAZF1* intron-3 and *SUZ12* intron-1, respectively. The pGEM-T plasmid was used for cloning the genomic DNA PCR product.
(TIF)

S8 Fig Sanger sequencing of the genomic breakpoint ‘y’ confirmed that the induced *JAZF1-SUZ12* fusion is the result of genomic rearrangement. hESC cells transfected with asJS-8 were enriched as described in S5 Fig. Genomic DNA PCR performed on the enriched hESC population yielded a second gene fusion band “y” of ~976 bp as shown in Fig 4D of the main text. Sanger sequencing confirmed that the 976 bp band contains ~491 bp of *JAZF1* intron-3 joined to ~483 bp of *SUZ12* intron-1. The genomic breakpoint between *JAZF1* intron-3 and *SUZ12* intron-1 is indicated by the dashed vertical lines that contains a two nucleotide ‘AA’ insertion. The locations of the primers used to amplify the fusion DNA product are marked as the light blue and dark blue arrows in *JAZF1* intron-3 and *SUZ12* intron-1, respectively. The pGEM-T plasmid was used for cloning the genomic DNA PCR product.
(TIF)

S1 Text List of primers used. (DOCX)

S2 Text Chimeric RNA sequences. (DOCX)

S3 Text Sequences for JAZF1 and SUZ12 genomic regions constituting the genomic stem. (DOCX)

S4 Text Genomic coordinates (UCSC Blat-hg38) for JAZF1 and SUZ12 sequences constituting the genomic stems. (DOCX)
