# Supplementary figures and images for: RNA-driven JAZF1-SUZ12 gene fusion in human endometrial stromal cells
Source: PLoS Genet. 2021 Dec 20;17(12):e1009985. doi: 10.1371/journal.pgen.1009985 (PMC8722726; doi:10.1371/journal.pgen.1009985)

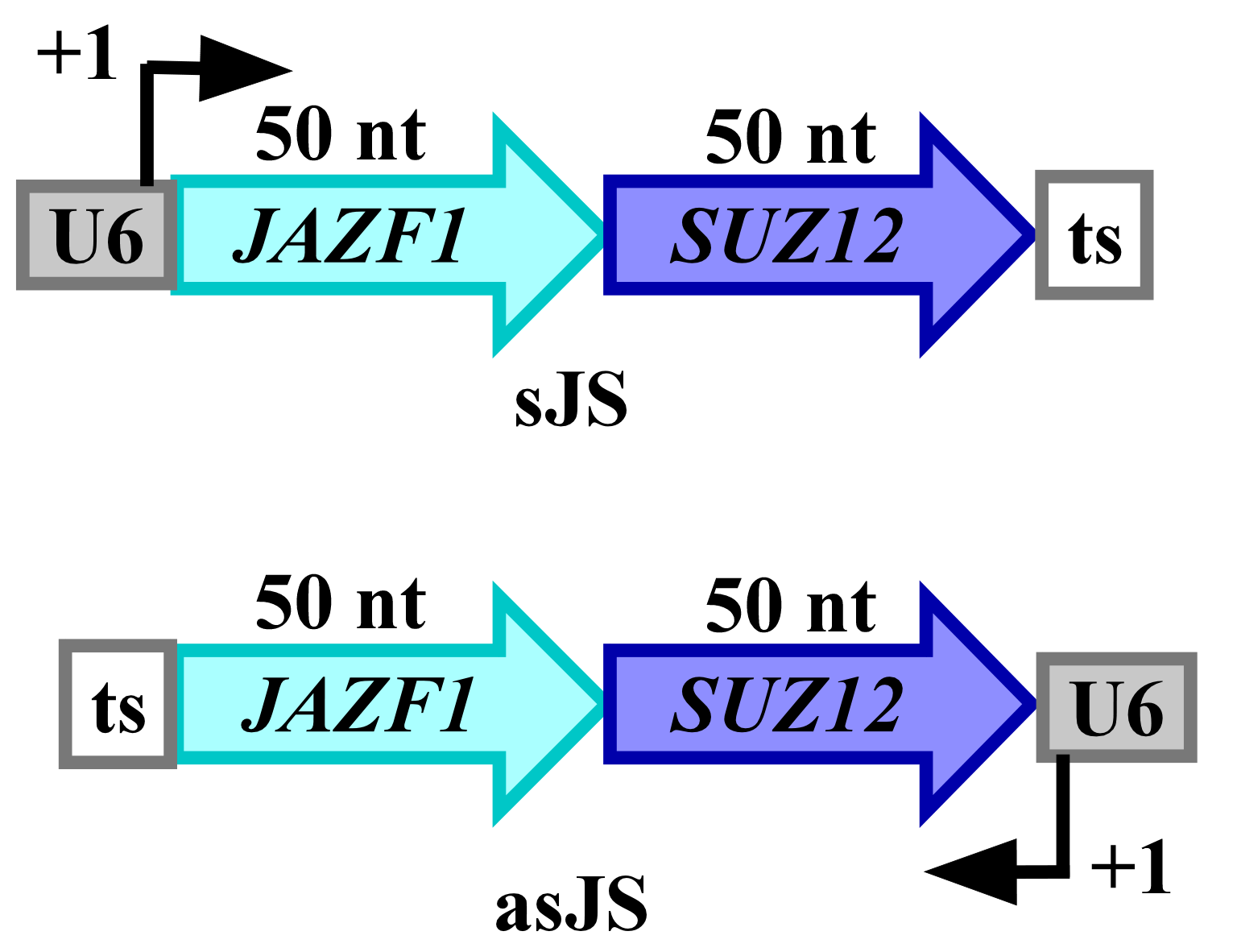

Supplement: S3 Fig — The chimeric RNAs are designed with 50 nts targeting JAZF1 gene and 50 nts targeting SUZ12 gene, and expressed either in the sense or antisense orientation using the U6 promoter. Plasmid expressing sense chimeric RNA contains the same DNA sequences as the plasmid expressing antisense chimeric RNA except that the U6 promoter is placed in the opposite direction. ‘ts’ represents the transcriptional stop sequence “TTTTTT” for U6 promoter. (TIF) [file pgen.1009985.s003.tif]

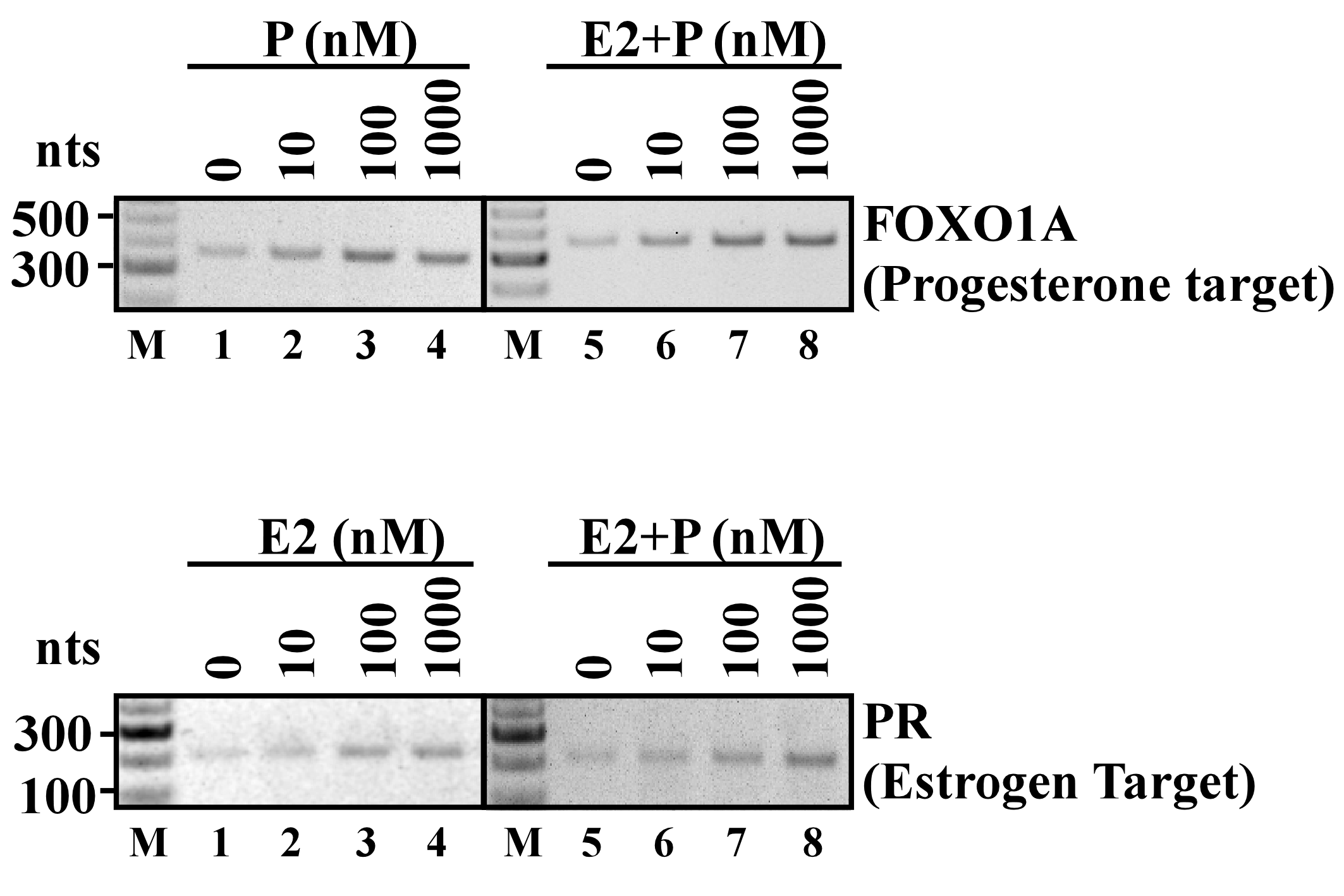

Supplement: S4 Fig — The hESC cells were treated with different concentrations (0nM, 10nM, 100nM and 1μM) of either estrogen (E2) or progesterone (P) or both (E2 + P) for three days. RT-PCR was performed for FOXO1A (a progesterone target gene, upper panel) and PR (an estrogen target gene, lower panel) respectively. The results indicate that hESC cells responded to estrogen and progesterone moderately at 10nM and more robustly at 100nM or 1μM. (TIF) [file pgen.1009985.s004.tif]

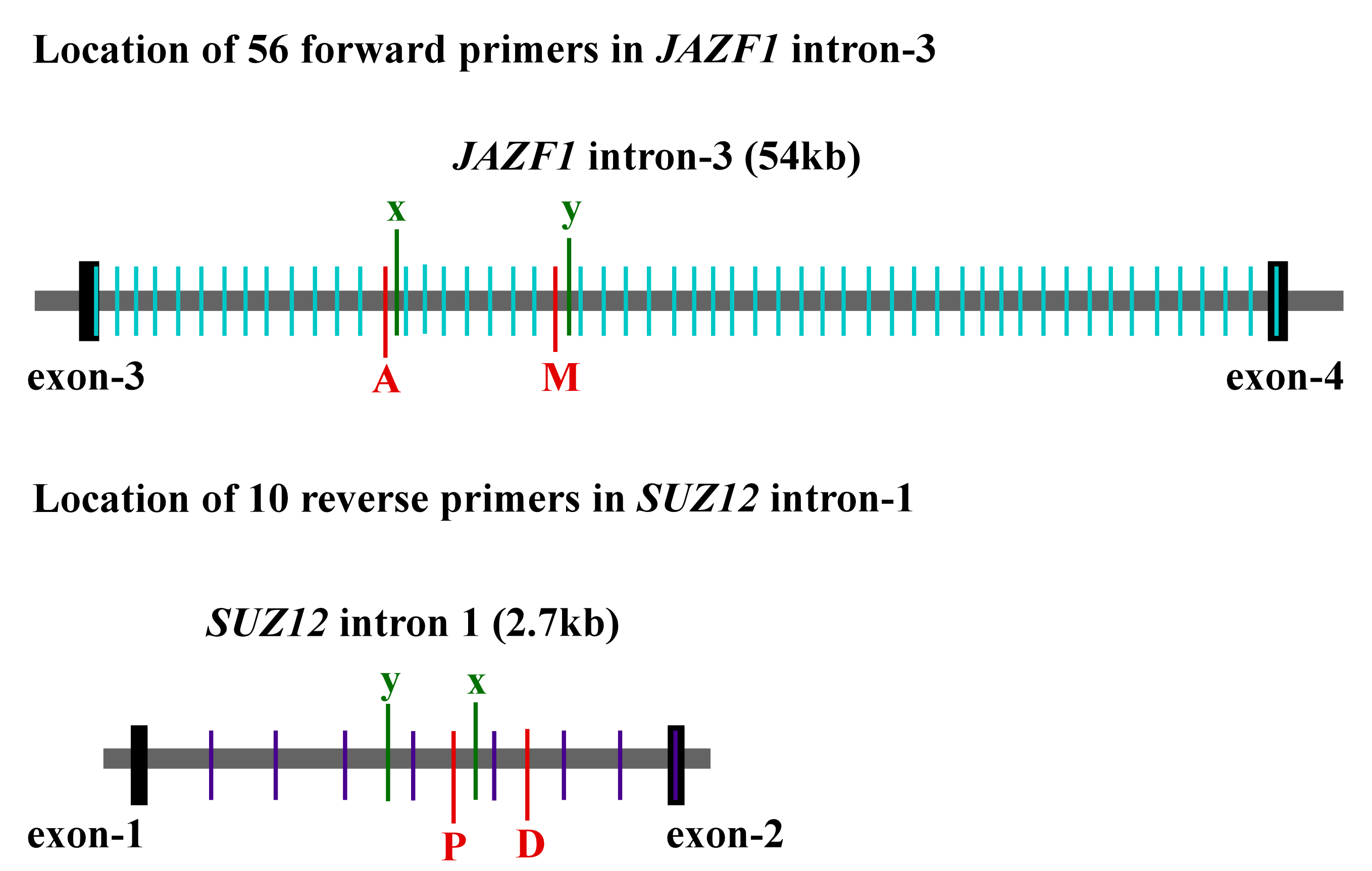

Supplement: S6 Fig — The locations of 56 forward primers (light blue) spacing across JAZF1 intron-3 (~54 kb) and 10 reverse primers (dark blue) spacing across SUZ12 intron-2 (~2.7 kb) are shown. Each vertical line represents a target location by a nested primer set. The locations of identified genomic breakpoint ‘x’ and ‘y’ are marked by dark green lines. The specific primers that initially identified the genomic breakpoint ‘x’ and ‘y’ are labeled as red. (TIF) [file pgen.1009985.s006.tif]
