# Supplementary material for: RNA-driven JAZF1-SUZ12 gene fusion in human endometrial stromal cells
Source: PLoS Genet. 2021 Dec 20;17(12):e1009985. doi: 10.1371/journal.pgen.1009985 (PMC8722726; doi:10.1371/journal.pgen.1009985)
Supplement: S1 Text — (DOCX) [file pgen.1009985.s009.docx]

**S1 Text. List of primers used**

**RT-PCR primers for amplifying induced fusion RNAs:**

| **Induced fusion RNA (*JAZF1*** **ex3-*SUZ12* ex2-3)** | |
| --- | --- |
| First round PCR | |
| *JAZF1* 3F1 | 5’**-** AAGATTCAGCCGAAGCTCTCG -3’ |
| *SUZ12* 3R1 | 5’- TGTTTGTTCTGGAGTTTCGATGAGACA -3’ |
| Second round PCR | |
| *JAZF1* 3F2 | 5’- TGTCCAGCTCAGTGTCTCGA-3’ |
| *SUZ12* 3R2 | 5’- CATGTAAGTAAGAGTTCTGT-3’ |
| Third round PCR | |
| *JAZF1* 3F3 | 5’- TGTCCACTCCCCCACGCCACA-3’ |
| *SUZ12* 3R2 | 5’- CATGTAAGTAAGAGTTCTGT-3’ |

| **Induced fusion RNA (*TMPRSS2* ex1-*ERG* ex4)** | |
| --- | --- |
| *TMPRSS2* ex-1 F1 | 5’**-** TAGGCGCGAGCTAAGCAGGAG -3’ |
| *ERG* ex-4 R1 | 5’**-** CTTGAGCCATTCACCTGGCTAG-3’ |

**PCR primers used for amplifying the GAPDH RNA**

| ***GAPDH*** | |
| --- | --- |
| *GAPDH* F1 | 5’- GCGTCTTCACCACCATGGAGA -3’ |
| *GAPDH* R1 | 5’**-** AGCCTTGGCAGCGCCAGTAGA -3’ |

**PCR primers used for amplifying the Chimeric RNA**

| **sJS*-*8*/* aJS-8** | |
| --- | --- |
| JS-8 F1 | 5’- TAGCTGCAGAATAAATATGCATTACAG -3’ |
| JS-8 R1 | 5’**-** ACGAAGCTTAAAAAATCAACATAAGAACTATTGA  TTGATGCCAAACAAGATGATAATTTCACTTT -3’ |
| **sJS*-*14*/* aJS-14** | |
| JS-14 F1 | 5’- TAGCTGCAGCTCATTGAGTTAATGTA -3’ |
| JS-14 R1 | 5’**-** ACGAAGCTTAAAAAACGAATAATCTCATTCCTC -3’ |
| **sJS-53/ aJS-53** | |
| JS-53 F1 | 5’- TAGCTGCAGCGACAGTACCATCATGCCTT  GAGTGTTCTTATCTCCCAAGTGCTATTCCT -3’ |
| JS-53 R1 | 5’**-** ACGAAGCTTAAAAAATTGCAAACTTATTTTATAAT -3’ |

**PCR primers used for amplifying endogenous mRNA**

| ***JAZF1*** | |
| --- | --- |
| *JAZF1* 3F3 | 5’- TGTCCACTCCCCCACGCCACA-3’ |
| *JAZF1* 5R1 | 5’**-** GATGGAAATTGATTGTGTGGT-3’ |

**PCR primers used for amplifying FOXO1A mRNA**

| ***FOXO1A*** | |
| --- | --- |
| *FOXO1A F1* | 5’- CTGGCTCTCACAGCAATGAT -3’ |
| *FOXO1A R2* | 5’**-** CTGGGTGAATTCAAACTGGT -3’ |

**PCR primers used for amplifying PR mRNA**

| ***PR*** | |
| --- | --- |
| *PR F2* | 5’- GTCCACAGCTGTCACTAATC -3’ |
| *PR R1* | 5’**-** TACAGCCCATTCCCAGGAAG -3’ |

**PCR primers used for amplifying the identified JAZF1-SUZ12 genomic DNA breakpoint:**

| **Breakpoint ‘x’** | |
| --- | --- |
| A’ (*JAZF1*-1F2) | 5’- ATGAAATTAATTGGTAAGATA -3’ |
| A (*JAZF1*-1F3) | 5’- TAAGATATAACCAGCATTTTA -3’ |
| B’ (*JAZF1-*1R1) | 5’- GCAAAACCCATTCTGGCAAGT -3’ |
| B (*JAZF1-*1R2) | 5’- AAGCCCTTGGAGACCAGCCCT -3’ |
| C’ (*SUZ12*-1F1) | 5’- TTAAGGAATGTTATATCCTGA -3’ |
| C (*SUZ12*-1F2) | 5’- TAATTTGGGTGATCTTTAATA -3’ |
| D’ (*SUZ12*-1R2) | 5’- TTAAAATGTAATTTGTTATAC -3’ |
| D (*SUZ12*-1R3) | 5’- AGTCGATGAAAAATACATATA -3’ |
| **Breakpoint ‘y’** | |
| M’ (*JAZF1*-2F1) | 5’- CCTCAGGACCCAACTTGTAAC -3’ |
| M (*JAZF1*-2F2) | 5’- TGTAACTTCTCGTTACAATGA -3’ |
| N’ (*JAZF1-*2R2) | 5’- ACCTCAGCCTCCCAAAGTGCT -3’ |
| N (*JAZF1-*2R3) | 5’- CACCATGCCTGGCCCCTAACT -3’ |
| O’ (*SUZ12*-2F2) | 5’- GGTTTCGGTTGTAGCTTTTGA -3’ |
| 0 (*SUZ12*-2F3) | 5’- AAAATGAAGCCTGTGAAGGGA -3’ |
| P’ (*SUZ12*-2R1) | 5’- AAAATTTCCACCTAAATCTTC -3’ |
| P (*SUZ12*-2R2) | 5’- TAACTAAAGGGAGATGAGATA -3’ |
