# Supplementary material for: RNA-driven JAZF1-SUZ12 gene fusion in human endometrial stromal cells
Source: PLoS Genet. 2021 Dec 20;17(12):e1009985. doi: 10.1371/journal.pgen.1009985 (PMC8722726; doi:10.1371/journal.pgen.1009985)
Supplement: S2 Text — (DOCX) [file pgen.1009985.s010.docx]

**S2 Text. Chimeric RNAs sequence**

**asJS-1**

**+1**tgtgctcgcttcggcagcacatatactaacattggaacgatcctgcagTCAACAAAAGAACTATTGATTGATGCCAAACAAGATGAAAATTTCACTT**A**TAAATATTGATTACAAACACACTCAAATTTGACATATTTCAACAAAATCA

G : *JAZF1* G: *SUZ12* **+1**: Transcription start ctgcag: PstI

**A**: T to A change to inactivate the cryptic transcription termination by U6 promoter

**asJS-2**

**+1**tgtgctcgcttcggcagcacatatactaacattggaacgatcctgcagTCAACAAAAGAACTATTGATTGATGCCAAACAAGATGAAAATTTCACTT**A**TGTTTGGGAAGGTT**A**TT**A**TCTAAGTGGTATAGCTGTGGAAAAACAAATAA

G : *JAZF1* G: *SUZ12* **+1**: Transcription start ctgcag: PstI

**A**: T to A change to inactivate the cryptic transcription termination by U6 promoter

**asJS-3**

**+1**tgtgctcgcttcggcagcacatatactaacattggaacgatcctgcagTCAACAAAAGAACTATTGATTGATGCCAAACAAGATGAAAATTTCACTTTAGGGTCATTTGAGGTTGCAACTCCCTCAGTGTGGCCAGCCCT**A**TTCTCAG

G : *JAZF1* G: *SUZ12* **+1**: Transcription start ctgcag: PstI

**A**: T to A change to inactivate the cryptic transcription termination by U6 promoter

**asJS-5**

**+1**tgtgctcgcttcggcagcacatatactaacattggaacgatcctgcagTCAACAAAAGAACTATTGATTGATGCCAAACAAGATGAAAATTTCACTTTGTT**A**TTGTAGACTGAACTTCAGGTGTGATATCCAAAAAATTCATTGACAA

G : *JAZF1* G: *SUZ12* **+1**: Transcription start ctgcag: PstI

**A**: T to A change to inactivate the cryptic transcription termination by U6 promoter

**asJS-6**

**+1**tgtgctcgcttcggcagcacatatactaacattggaacgatcctgcagTCAACAAAAGAACTATTGATTGATGCCAAACAAGATGAAAATTTCACTT**A**TGCCTCCAACTTTACTT**A**TTCTTTCTTAGGATCACTTTGGTTATTTAGGG

G : *JAZF1* G: *SUZ12* **+1**: Transcription start ctgcag: PstI

**A**: T to A change to inactivate the cryptic transcription termination by U6 promoter

**asJS-7**

**+1**tgtgctcgcttcggcagcacatatactaacattggaacgatcctgcagTCAACAAAAGAACTATTGATTGATGCCAAACAAGATGAAAATTTCACTT**A**TCCTGGGGTCTTGGAGGAGGTACTGGTCCTGCTGTGCCTCTCCCTCCAGC

G : *JAZF1* G: *SUZ12* **+1**: Transcription start ctgcag: PstI

**A**: T to A change to inactivate the cryptic transcription termination by U6 promoter

**asJS-8**

**+1**tgtgctcgcttcggcagcacatatactaacattggaacgatcctgcagTCAACAAAAGAACTATTGATTGATGCCAAACAAGATGAAAATTTCACTTTGCGTATTAGTTACCAAAGGTTTATTTATGTTTCTGTAATGCATATTTAT**A**

G : *JAZF1* G: *SUZ12* **+1**: Transcription start ctgcag: PstI

**A**: T to A change to inactivate the cryptic transcription termination by U6 promoter

**asJS-9**

**+1**tgtgctcgcttcggcagcacatatactaacattggaacgatcctgcagAATACATCCTAATACAGATATGATGACATCCTAATACAGATCCAGACATTGCTACACAGGAGGCTGACGTGAGCTACACAGGAGGCTGAGCCCAGGAGT**A**

G : *JAZF1* G: *SUZ12* **+1**: Transcription start ctgcag: PstI

**A**: T to A change to inactivate the cryptic transcription termination by U6 promoter

**asJS-10**

**+1**tgtgctcgcttcggcagcacatatactaacattggaacgatcctgcagAATACATCCTAATACAGATATGATGACATCCTAATACAGATCCAGACATTAGCTACTTGGGAGGCAGAGGTCAGAGAATCTCTTGAACCTGAAAGGTGGA

G : *JAZF1* G: *SUZ12* **+1**: Transcription start ctgcag: PstI

**asJS-11**

**+1**tgtgctcgcttcggcagcacatatactaacattggaacgatcctgcagAATACATCCTAATACAGATATGATGACATCCTAATACAGATCCAGACATTGCTACTCAGGAGGCTGAGGCAGGAGAATCGCTTGAACCCGGGAGGCAGAG

G : *JAZF1* G: *SUZ12* **+1**: Transcription start ctgcag: PstI

**asJS-12**

**+1**tgtgctcgcttcggcagcacatatactaacattggaacgatcctgcagTAATACAGATCCAGACATTCCTAATACAGGTATGATGAAAGTCAGTCAAGAGCCAGGTGCAGTAGCACACGCTTGTGATGCCAGCTACTCAGGAGGCTGA

G : *JAZF1* G: *SUZ12* **+1**: Transcription start ctgcag: PstI

**asJS-13**

**+1**tgtgctcgcttcggcagcacatatactaacattggaacgatcctgcagAATTGCCTGAACCCGGGAAGCGGAGGTTGCAGTGAGCCGATATCGCACCAATTGTT**A**TACTTCTTCCTTTCCAGTCTACCTTAATT**A**TGCCTTGATCCAC

G : *JAZF1* G: *SUZ12* **+1**: Transcription start ctgcag: PstI

**A**: T to A change to inactivate the cryptic transcription termination by U6 promoter

**asJS-14**

**+1**tgtgctcgcttcggcagcacatatactaacattggaacgatcctgcagCGAATAATCTCATTCCTCAAAGAGAGTT**A**TGTGTTTGTTTGTT**A**TTGTTTATACCTAAGTTGTAAGAGATTCACAGTTTACCCTACATTAACTCAATGAG

G : *JAZF1* G: *SUZ12* **+1**: Transcription start ctgcag: PstI

**A**: T to A change to inactivate the cryptic transcription termination by U6 promoter

**asJS-15**

**+1**tgtgctcgcttcggcagcacatatactaacattggaacgatcctgcagCAATTTATTACTTTATAATGCTTGAATGCAGACACCAAAAATTACGAATAAAAAGTTACCTACGTATATAGGTTTGAAAAGGTCTACTGTTCTCAGCTTC

G : *JAZF1* G: *SUZ12* **+1**: Transcription start ctgcag: PstI

**asJS-16**

**+1**tgtgctcgcttcggcagcacatatactaacattggaacgatcctgcagAAAAAAAGAGAAATAACCCATGGCACAGAGTAAAAATATAATCAGTAAAATTCACATATTATACAATTTGCCATTTAAAGTATACAATTTAGTGGTT**A**TT

G : *JAZF1* G: *SUZ12* **+1**: Transcription start ctgcag: PstI

**A**: T to A change to inactivate the cryptic transcription termination by U6 promoter

**asJS-17**

**+1**tgtgctcgcttcggcagcacatatactaacattggaacgatcctgcagATAATAATTTAATTACTTTGTAAATTAAGATCAGGTTGCATCCTAGGCAGCATTTATCCTCATACCATTGTGAATAAAGATTGTT**A**TACTTCTTCCTTTC

G : *JAZF1* G: *SUZ12* **+1**: Transcription start ctgcag: PstI

**A**: T to A change to inactivate the cryptic transcription termination by U6 promoter

**asJS-18**

**+1**tgtgctcgcttcggcagcacatatactaacattggaacgatcctgcagCAAACAAAAAAAGAGAAATAACCCATGGCACAGAGTAAAAATATAATCAGTAAAAGTTACCTACGTATATAGGTTTGAAAAGGTCTACTGTTCTCAGCT**A**

G : *JAZF1* G: *SUZ12* **+1**: Transcription start ctgcag: PstI

**A**: T to A change to inactivate the cryptic transcription termination by U6 promoter

**asJS-19**

**+1**tgtgctcgcttcggcagcacatatactaacattggaacgatcctgcagTATAATCAGTAAAATCACTTTATTCACTTCACTACTAAAATCAGTT**A**TGTATACCTAAGTTGTAAGAGATTCACAGTTTACCCTACATTAACTCAATGAG

G : *JAZF1* G: *SUZ12* **+1**: Transcription start ctgcag: PstI

**A**: T to A change to inactivate the cryptic transcription termination by U6 promoter

**asJS-20**

**+1**tgtgctcgcttcggcagcacatatactaacattggaacgatcctgcagATCTGGGAGGCAGAGGTTGCAGTGAGCCGAGGTTGCGCCACTGCACTCCATTATCCTCATACCATTGTGAATAAAGATTGTT**A**TACTTCTTCCTTTCCAG

G : *JAZF1* G: *SUZ12* **+1**: Transcription start ctgcag: PstI

**A**: T to A change to inactivate the cryptic transcription termination by U6 promoter

**asJS-21**

**+1**tgtgctcgcttcggcagcacatatactaacattggaacgatcctgcagTTTACGTAACAAATATAGTTACCCTGAGCCCTTTAGTAAATATTTAAATCTACATTTATCCTCATACCATTGTGAATAAAGATTGTT**A**TACTTCTTCCT**A**

G : *JAZF1* G: *SUZ12* **+1**: Transcription start ctgcag: PstI

**A**: T to A change to inactivate the cryptic transcription termination by U6 promoter

**asJS-22**

**+1**tgtgctcgcttcggcagcacatatactaacattggaacgatcctgcagATCCTGCAATGGGACAAATTAATAAACATGACGGGAAAATGGAACTTAGAATT**A**TTTGGGATT**A**TTTACATTTATCCTCATACCATTGTGAATAAAGAT**A**

G : *JAZF1* G: *SUZ12* **+1**: Transcription start ctgcag: PstI

**A**: T to A change to inactivate the cryptic transcription termination by U6 promoter

**asJS-23**

**+1**tgtgctcgcttcggcagcacatatactaacattggaacgatcctgcagATTACTTTATAATGCTTGAATGCAGACACCAAAAATTACGAATAATCTCATTCACATATTATACAATTTGCCATTTAAAGTATACAATTTAGTGGT**A**TT**A**

G : *JAZF1* G: *SUZ12* **+1**: Transcription start ctgcag: PstI

**A**: T to A change to inactivate the cryptic transcription termination by U6 promoter

**asJS-24**

**+1**tgtgctcgcttcggcagcacatatactaacattggaacgatcctgcagTAATGATTTAAGACTTCATTGTT**A**TTAAAAAACCTTCATCCTATTGATT**A**TACATTTATCCTCATACCATTGTGAATAAAGATTGTT**A**TACTTCTTCCT**A**

G : *JAZF1* G: *SUZ12* **+1**: Transcription start ctgcag: PstI

**A**: T to A change to inactivate the cryptic transcription termination by U6 promoter

**asJS-25**

**+1**tgtgctcgcttcggcagcacatatactaacattggaacgatcctgcagATAATAATTTAATTACTTTGTAAATTAAGATCAGGTTGCATCCTAGGCAGTTGTCCAGGCTTGTCTCAAACAGGCAATCCTCCTGCTTTGGCCTCCCAAG

G : *JAZF1* G: *SUZ12* **+1**: Transcription start ctgcag: PstI

**asJS-26**

**+1**tgtgctcgcttcggcagcacatatactaacattggaacgatcctgcagCAAACAAAAAAAGAGAAATAACCCATGGCACAGAGTAAAAATATAATCAGGCTT**A**TAATAAGAAAACAAGAATGTT**A**TTGTACAGAGTATATCCTGAGCA

G : *JAZF1* G: *SUZ12* **+1**: Transcription start ctgcag: PstI

**A**: T to A change to inactivate the cryptic transcription termination by U6 promoter

**asJS-27**

**+1**tgtgctcgcttcggcagcacatatactaacattggaacgatcctgcagAGCTACTAAGGAGGCTGAAGGAGGAGAATCTCTTGAATCTGGGAGGCAGACAAGTGCCACATTGTGAAAATCAGTGAGAAACAGGAAATGAAGGTGGCT**A**

G : *JAZF1* G: *SUZ12* **+1**: Transcription start ctgcag: PstI

**A**: T to A change to inactivate the cryptic transcription termination by U6 promoter

**asJS-28**

**+1**tgtgctcgcttcggcagcacatatactaacattggaacgatcctgcagGTGAGCCGATATCGCACCACTGCACTCCAGCCTGGGAGACAAGAGCGAAACACTATGTTGTCCAGGCTTGTCTCAAACAGGCAATCCTCCTGCTTTGGCC

G : *JAZF1* G: *SUZ12* **+1**: Transcription start ctgcag: PstI

**asJS-29**

**+1**tgtgctcgcttcggcagcacatatactaacattggaacgatcctgcagTTTACGTAACAAATATAGTTACCCTGAGCCCTTTAGTAAATATTTAAATCTGTTGTCCAGGCTTGTCTCAAACAGGCAATCCTCCTGCTTTGGCCTCCCA

G : *JAZF1* G: *SUZ12* **+1**: Transcription start ctgcag: PstI

**asJS-30**

**+1**tgtgctcgcttcggcagcacatatactaacattggaacgatcctgcagACATCAATTTATTACTTTATAATGCTTGAATGCAGACACCAAAAATTACGTAATAAGAAAACAAGAATGTT**A**TTGTACAGAGTATATCCTGAGCAGATAA

G : *JAZF1* G: *SUZ12* **+1**: Transcription start ctgcag: PstI

**A**: T to A change to inactivate the cryptic transcription termination by U6 promoter

**asJS-31**

**+1**tgtgctcgcttcggcagcacatatactaacattggaacgatcctgcagTAATGATTTAAGACTTCATTGTT**A**TTAAAAAACCTTCATCCTATTGATT**A**TGTTGTCCAGGCTTGTCTCAAACAGGCAATCCTCCTGCTTTGGCCTCCCA

G : *JAZF1* G: *SUZ12* **+1**: Transcription start ctgcag: PstI

**A**: T to A change to inactivate the cryptic transcription termination by U6 promoter

**asJS-32**

**+1**tgtgctcgcttcggcagcacatatactaacattggaacgatcctgcagATCCTGCAATGGGACAAATTAATAAACATGACGGGAAAATGGAACTTAGAAGATAGGGTCTCACTATGTTGTCCAGGCTTGTCTCAAACAGGCAATCCTC

G : *JAZF1* G: *SUZ12* **+1**: Transcription start ctgcag: PstI

**asJS-33**

**+1**tgtgctcgcttcggcagcacatatactaacattggaacgatcctgcagAGTTGTGCTTTAATTTCTTAATAATTAGTCCTAATAAGTAATAAGATCAGAAAGTTACCTACGTATATAGGTTTGAAAAGGTCTACTGTTCTCAGCTTCA

G : *JAZF1* G: *SUZ12* **+1**: Transcription start ctgcag: PstI

**asJS-34**

**+1**tgtgctcgcttcggcagcacatatactaacattggaacgatcctgcagTGCTTTAATTTCTTAATAATTAGTCCTAATAAGTAATAAGATCAGGCATCCACATATTATACAATTTGCCATTTAAAGTATACAATTTAGTGGTT**A**TTAA

G : *JAZF1* G: *SUZ12* **+1**: Transcription start ctgcag: PstI

**A**: T to A change to inactivate the cryptic transcription termination by U6 promoter

**asJS-35**

**+1**tgtgctcgcttcggcagcacatatactaacattggaacgatcctgcagTGAGTTGTGCTTTAATTTCTTAATAATTAGTCCTAATAAGTAATAAGATCTTAATAAGAAAACAAGAATGTT**A**TTGTACAGAGTATATCCTGAGCAGATA

G : *JAZF1* G: *SUZ12* **+1**: Transcription start ctgcag: PstI

**A**: T to A change to inactivate the cryptic transcription termination by U6 promoter

**asJS-36**

**+1**tgtgctcgcttcggcagcacatatactaacattggaacgatcctgcagCACTTCAGGAGGCCACGGTGAGTGGATCACCTGAGGTCAGGAGTTCAAGAACAGGGTCTTACTCTGTCACCCAGGCTGGAGTGCAGTGGTGCCATCATGG

G : *JAZF1* G: *SUZ12* **+1**: Transcription start ctgcag: PstI

**asJS-38**

**+1**tgtgctcgcttcggcagcacatatactaacattggaacgatcctgcagAGGTCAGGAGTTTGAGACCAGCCTGACCAACATGGTGAAACCCTGTCTCTAAGTCAAAAAAATGTT**A**TGTTTGGGAAGGTT**A**TTTTCTAAGTGGTATAGC

G : *JAZF1* G: *SUZ12* **+1**: Transcription start ctgcag: PstI

**A**: T to A change to inactivate the cryptic transcription termination by U6 promoter

**asJS-39**

**+1**tgtgctcgcttcggcagcacatatactaacattggaacgatcctgcagAAAAATATAATCAGTAAAATCACTTTATTCACTTCACTACTAAAATCAGTTAGCCAGGTGCAGTAGCACACGCTTGTGATGCCAGCTACTCAGGAGGCTG

G : *JAZF1* G: *SUZ12* **+1**: Transcription start ctgcag: PstI

**asJS-40**

**+1**tgtgctcgcttcggcagcacatatactaacattggaacgatcctgcagAATTACGAATAATCTCATTCCTCAAAGAGAGTT**A**TGTGTTTGTTTGTT**A**TATTAAGTGGGCATTGTGGCATGTGCCTGTAGTCCCAGCTACACAGGAGGC

G : *JAZF1* G: *SUZ12* **+1**: Transcription start ctgcag: PstI

**A**: T to A change to inactivate the cryptic transcription termination by U6 promoter

**asJS-41**

**+1**tgtgctcgcttcggcagcacatatactaacattggaacgatcctgcagGCAATGGGACAAATTAATAAACATGACGGGAAAATGGAACTTAGATT**A**TATAGTAGAGACAGGGTTTCACCATGTTGGTCAGGCTGGTCTTGAACCCCTA

G : *JAZF1* G: *SUZ12* **+1**: Transcription start ctgcag: PstI

**A**: T to A change to inactivate the cryptic transcription termination by U6 promoter

**asJS-42**

**+1**tgtgctcgcttcggcagcacatatactaacattggaacgatcctgcagCCAGCTACTCGGGAGGCTGAGGCAGGAGAATCACTTGAACCCAGGAGGTGGGATTACAGGTATGAGCCACGGTGCCTGGCCATT**A**TAAAATT**A**TTTATTG

G : *JAZF1* G: *SUZ12* **+1**: Transcription start ctgcag: PstI

**A**: T to A change to inactivate the cryptic transcription termination by U6 promoter

**asJS-43**

**+1**tgtgctcgcttcggcagcacatatactaacattggaacgatcctgcagTTTCATT**A**TCTCTACTT**A**TGATAACATATCATCAACTTTAAATGTT**A**TCCTTTAAAAGCTTCATTTCCAAATTAAAGTATT**A**TTAATCTACTGTGGTTT**A**

G : *JAZF1* G: *SUZ12* **+1**: Transcription start ctgcag: PstI

**A**: T to A change to inactivate the cryptic transcription termination by U6 promoter

**asJS-44**

**+1**tgtgctcgcttcggcagcacatatactaacattggaacgatcctgcagCATT**A**TCTCTACTT**A**TGATAACATATCATCAACTTTAAATGTT**A**TCCTGGGGATGTTGAACTGCATTTGGTT**A**TTGGATGTTAAAATGACTT**A**TT**A**TGTG

G : *JAZF1* G: *SUZ12* **+1**: Transcription start ctgcag: PstI

**A**: T to A change to inactivate the cryptic transcription termination by U6 promoter

**asJS-45**

**+1**tgtgctcgcttcggcagcacatatactaacattggaacgatcctgcagATATCACTTAATGTAAACTGATAGATTATCTTACCTATGAGATTCTTGCTCGTGGTATATGACAAATGCAGATGCTCCTCAACTTACGATGGGGTTACAT

G : *JAZF1* G: *SUZ12* **+1**: Transcription start ctgcag: PstI

**asJS-46**

**+1**tgtgctcgcttcggcagcacatatactaacattggaacgatcctgcagCCCCTACCCTCACAAAAATAAAATTCTCATTTCTCATTTGTAGGCCTGTATT**A**TATTCTCAGAAGCGTCTGTTACTCCAAAATGATTGAGAACCAGTGC**A**

G : *JAZF1* G: *SUZ12* **+1**: Transcription start ctgcag: PstI

**A**: T to A change to inactivate the cryptic transcription termination by U6 promoter

**asJS-47**

**+1**tgtgctcgcttcggcagcacatatactaacattggaacgatcctgcagACCTATGAGATTCTTGCTCTCCTTTCATT**A**TCTCTACTT**A**TGATAACATACCTAAACATT**A**TATCTATTGATT**A**TACTCTAAGTAGAATTATTAAAAATA

G : *JAZF1* G: *SUZ12* **+1**: Transcription start ctgcag: PstI

**A**: T to A change to inactivate the cryptic transcription termination by U6 promoter

**asJS-48**

**+1**tgtgctcgcttcggcagcacatatactaacattggaacgatcctgcagATACGCTCAAATAAATATAATCTTGCACCCAGGACAAAAAACTTGCATTCTCGTGCCACTGCACTCCTGGGTGACAGAGCAAGACCTTGTCTCAAAAAAA

G : *JAZF1* G: *SUZ12* **+1**: Transcription start ctgcag: PstI

**asJS-49**

**+1**tgtgctcgcttcggcagcacatatactaacattggaacgatcctgcagTGGGAGACAGAGCGAGACTCCGTTTCAAAAAAAAAAAGGCCAGGCACGGTATCACACCCAGCTAATT**A**TTGTATTTCATGTAGAGAAAGGGTTTGCCATG

G : *JAZF1* G: *SUZ12* **+1**: Transcription start ctgcag: PstI

**A**: T to A change to inactivate the cryptic transcription termination by U6 promoter

**asJS-50**

**+1**tgtgctcgcttcggcagcacatatactaacattggaacgatcctgcagTAAAAACAAAACAAAAAAAAAACCAAAGATATAAAATGAGACTTTCTGAAGCAAAATTATT**A**TTAATT**A**TTGAATATGGAGCAAAGTAAAACTT**A**TGAAA

G : *JAZF1* G: *SUZ12* **+1**: Transcription start ctgcag: PstI

**A**: T to A change to inactivate the cryptic transcription termination by U6 promoter

**asJS-51**

**+1**tgtgctcgcttcggcagcacatatactaacattggaacgatcctgcagAGGCGGAGCTTGCAGTGAGCTGAGATCACGCCACTGCACTCCAACCTGGATTACTTCTTTGGGGAAAGTGGAATTACTAGGTCAGTTCCACTAGCAACTG

G : *JAZF1* G: *SUZ12* **+1**: Transcription start ctgcag: PstI

**asJS-52**

**+1**tgtgctcgcttcggcagcacatatactaacattggaacgatcctgcagATTGCCTGAACCCGGGAAGCGGAGGTTGCAGTGAGCCGATATCGCACCACATTTCTTATTCTCTGGCAGCAACACAATACTGATGATAATTGAATTAGT**A**

G : *JAZF1* G: *SUZ12* **+1**: Transcription start ctgcag: PstI

**A**: T to A change to inactivate the cryptic transcription termination by U6 promoter

**asJS-53**

**+1**tgtgctcgcttcggcagcacatatactaacattggaacgatcctgcagATGCAAACTTATT**A**TATAATAATTTAATTACTTTGTAAATTAAGATCAGGAGGAATAGCACTTGGGAGAAAAGAACACTCAAGGCATGATGGTACTGTCG

G : *JAZF1* G: *SUZ12* **+1**: Transcription start ctgcag: PstI

**A**: T to A change to inactivate the cryptic transcription termination by U6 promoter

**sJS-1**

**+1**tgtgctcgcttcggcagcacatatactaacattggaacgatcctgcagTGATT**A**TGTTGAAATATGTCAAATTTGAGTGTGTTTGTAATCAATATTTAAAAGTGAAATT**A**TCATCTTGTTTGGCATCAATCAATAGTTCTT**A**TGTTGA

G : *JAZF1* G: *SUZ12* **+1**: Transcription start ctgcag: PstI

**A**: T to A change to inactivate the cryptic transcription termination by U6 promoter

**sJS-2**

**+1**tgtgctcgcttcggcagcacatatactaacattggaacgatcctgcagTTATTTGTT**A**TTCCACAGCTATACCACTTAGAtAAtAACCTTCCCAAACAAAAGTGAAATT**A**TCATCTTGTTTGGCATCAATCAATAGTTCTT**A**TGTTGA

G : *JAZF1* G: *SUZ12* **+1**: Transcription start ctgcag: PstI

**A**: T to A change to inactivate the cryptic transcription termination by U6 promoter

**sJS-8**

**+1**tgtgctcgcttcggcagcacatatactaacattggaacgatcctgcagAATAAATATGCATTACAGAAACATAAATAAACCTTTGGTAACTAATACGCAAAGTGAAATT**A**TCATCTTGTTTGGCATCAATCAATAGTTCTT**A**TGTTGA

G : *JAZF1* G: *SUZ12* **+1**: Transcription start ctgcag: PstI

**A**: T to A change to inactivate the cryptic transcription termination by U6 promoter

**sJS-11**

**+1**tgtgctcgcttcggcagcacatatactaacattggaacgatcctgcagCTCTGCCTCCCGGGTTCAAGCGATTCTCCTGCCTCAGCCTCCTGAGTAGCAATGTCTGGATCTGTATTAGGATGTCATCATATCTGTATTAGGATGTAT**A**

G : *JAZF1* G: *SUZ12* **+1**: Transcription start ctgcag: PstI

**A**: T to A change to inactivate the cryptic transcription termination by U6 promoter

**sJS-14**

**+1**tgtgctcgcttcggcagcacatatactaacattggaacgatcctgcagCTCATTGAGTTAATGTAGGGTAAACTGTGAATCTCTTACAACTTAGGTATAAACAATAACAAACAAACACATAACTCTCTTTGAGGAATGAGATTATTCG

G : *JAZF1* G: *SUZ12* **+1**: Transcription start ctgcag: PstI

**sJS-31**

**+1**tgtgctcgcttcggcagcacatatactaacattggaacgatcctgcagTGGGAGGCCAAAGCAGGAGGATTGCCTGTTTGAGACAAGCCTGGACAACATAATCAATAGGATGAAGGTT**A**TTTAAAAACAATGAAGTCTTAAATCATTA

G : *JAZF1* G: *SUZ12* **+1**: Transcription start ctgcag: PstI

**A**: T to A change to inactivate the cryptic transcription termination by U6 promoter

**sJS-36**

**+1**tgtgctcgcttcggcagcacatatactaacattggaacgatcctgcagCCATGATGGCACCACTGCACTCCAGCCTGGGTGACAGAGTAAGACCCTGTTCTTGAACTCCTGACCTCAGGTGATCCACTCACCGTGGCCTCCTGAAGTG

G : *JAZF1* G: *SUZ12* **+1**: Transcription start ctgcag: PstI

**sJS-38**

**+1**tgtgctcgcttcggcagcacatatactaacattggaacgatcctgcagGCTATACCACTTAGAAAAAAACCTTCCCAAACAAAACATT**A**TTTTGACTTAGAGACAGGGTTTCACCATGTTGGTCAGGCTGGTCTCAAACTCCTGACCT

G : *JAZF1* G: *SUZ12* **+1**: Transcription start ctgcag: PstI

**A**: T to A change to inactivate the cryptic transcription termination by U6 promoter

**sJS-39**

**+1**tgtgctcgcttcggcagcacatatactaacattggaacgatcctgcagCAGCCTCCTGAGTAGCTGGCATCACAAGCGTGTGCTACTGCACCTGGCTAACTGATT**A**TAGTAGTGAAGTGAATAAAGTGATT**A**TACTGATTATATTT**A**T

G : *JAZF1* G: *SUZ12* **+1**: Transcription start ctgcag: PstI

**A**: T to A change to inactivate the cryptic transcription termination by U6 promoter

**sJS-44**

**+1**tgtgctcgcttcggcagcacatatactaacattggaacgatcctgcagCACAAAAAAAGTCATT**A**TAACATCCAAAAACCAAATGCAGTTCAACATCCCCAGGATAACATTTAAAGTTGATGATATGTTATCAAAAGTAGAGAAAATG

G : *JAZF1* G: *SUZ12* **+1**: Transcription start ctgcag: PstI

**A**: T to A change to inactivate the cryptic transcription termination by U6 promoter

**sJS-45**

**+1**tgtgctcgcttcggcagcacatatactaacattggaacgatcctgcagATGTAACCCCATCGTAAGTTGAGGAGCATCTGCATTTGTCATATACCACGAGCAAGAATCTCATAGGTAAGATAATCTATCAGTTTACATTAAGTGATA**A**

G : *JAZF1* G: *SUZ12* **+1**: Transcription start ctgcag: PstI

**A**: T to A change to inactivate the cryptic transcription termination by U6 promoter

**sJS-51**

**+1**tgtgctcgcttcggcagcacatatactaacattggaacgatcctgcagCAGTTGCTAGTGGAACTGACCTAGTAATTCCACTTTCCCCAAAGAAGTAATCCAGGTTGGAGTGCAGTGGCGTGATCTCAGCTCACTGCAAGCTCCGCC**A**

G : *JAZF1* G: *SUZ12* **+1**: Transcription start ctgcag: PstI

**A**: T to A change to inactivate the cryptic transcription termination by U6 promoter

**sJS-53**

**+1**tgtgctcgcttcggcagcacatatactaacattggaacgatcctgcagCGACAGTACCATCATGCCTTGAGTGTTCTT**A**TCTCCCAAGTGCTATTCCTCCTGATCTTAATTTACAAAGTAATTAAATTATTATAAAATAAGTTTGCA**A**

G : *JAZF1* G: *SUZ12* **+1**: Transcription start ctgcag: PstI

**A**: T to A change to inactivate the cryptic transcription termination by U6 promoter
