# Supplementary material for: RNA-driven JAZF1-SUZ12 gene fusion in human endometrial stromal cells
Source: PLoS Genet. 2021 Dec 20;17(12):e1009985. doi: 10.1371/journal.pgen.1009985 (PMC8722726; doi:10.1371/journal.pgen.1009985)
Supplement: S3 Text — (DOCX) [file pgen.1009985.s011.docx]

**S3 Text. Sequences for JAZF1 and SUZ12 genomic regions constituting the genomic stem**

**Genomic stem-1**

| **Score** | **Expect** | **Identities** | **Gaps** | **Strand** |
| --- | --- | --- | --- | --- |
| 314 bits(347) | 4e-90 | 243/286(85%) | 5/286(1%) | Plus/Minus |

JAZF1 1 GTGGCTCACGCCTGTAATCCCAACACTTTGGGAGGCCGAGGCGGGTGGATCACCTAAGGT 60

|||||||| ||||||| ||||| |||||||||||||| ||| ||| |||||||||||||

SUZ12 285 GTGGCTCATGCCTGTATTCCCAGCACTTTGGGAGGCCAAGGTGGGCAGATCACCTAAGGT 226

JAZF1 61 TAGGGGTTCAAGACCAGCCTGACCAACATGGTGAAACCCTGTCTCTACTAAAAATACAAA 120

|| |||| ||||||||||||||||||||| ||||||||||||| || ||| |||||||

SUZ12 225 CGGGAGTTCGAGACCAGCCTGACCAACATGGAGAAACCCTGTCTCCACCAAATATACAAA 166

JAZF1 121 AACTAGCCAGGC---GTGGTGGCCGGTGCCTGTAATCCCAGCTACTCAGGAGGCTTAGGC 177

||| |||||||| | || ||| |||||||||||||||||||| ||||||| ||||

SUZ12 165 AAC-AGCCAGGCATAGGGGCGGCGCATGCCTGTAATCCCAGCTACTTGGGAGGCTGAGGC 107

JAZF1 178 AGGAGAATCATTTGAACCCGGGAGGTGGAAGTTGCAGTGAGCCAAGATCGTGCCATTGCA 237

|||||||||| ||||||| |||||| ||| | ||| ||||||| |||||| | ||||| |

SUZ12 106 AGGAGAATCACTTGAACCTGGGAGGCGGAGGCTGCCGTGAGCCGAGATCGCGTCATTGTA 47

JAZF1 238 CTCCAGTCTGGGCAA-CAAGAGTGAAACTCCATCTCaaaaaaaaaa 282

|||||| |||||||| ||||| |||||| | ||||||||||||||

SUZ12 46 CTCCAGCCTGGGCAATAAAGAGCGAAACTTCGTCTCAAAAAAAAAA 1

**Genomic stem -2**

| **Score** | **Expect** | **Identities** | **Gaps** | **Strand** |
| --- | --- | --- | --- | --- |
| 294 bits(325) | 4e-84 | 249/302(82%) | 6/302(1%) | Plus/Minus |

JAZF1 1 GGCCAGGCACAGTGGTTCACGCCTGTAATCCCAGCACTTTGGGAGTCCAAGACGGACAGA 60

|||||||| |||||| ||| ||||||| ||||||||||||||||| ||||| || ||||

SUZ12 301 GGCCAGGCGCAGTGGCTCATGCCTGTATTCCCAGCACTTTGGGAGGCCAAGGTGGGCAGA 242

JAZF1 61 TCCCCTGAGGTCAGGAGTTCGAGACCAGCCTGGCAAACATGGCAAAACCCCATCTCTACT 120

|| ||| ||||| ||||||||||||||||||| | ||||||| |||||| |||| ||

SUZ12 241 TCACCTAAGGTCGGGAGTTCGAGACCAGCCTGACCAACATGGAGAAACCCTGTCTCCACC 182

JAZF1 121 AAAAATACAAAAATTAGCCAGGCAT-GTTGGTGCGCA--CCTGTAATCCCAGCTACTTGG 177

||| ||||||||| |||||||||| | | ||||| |||||||||||||||||||||

SUZ12 181 AAATATACAAAAA-CAGCCAGGCATAGGGGCGGCGCATGCCTGTAATCCCAGCTACTTGG 123

JAZF1 178 GAGGCTGAGGCATGAGAATCAGTTGGACCTGGGAGGCAGAGGTTGTAGTGAGCCAAGATT 237

|||||||||||| |||||||| ||| ||||||||||| |||| || ||||||| ||||

SUZ12 122 GAGGCTGAGGCAGGAGAATCACTTGAACCTGGGAGGCGGAGGCTGCCGTGAGCCGAGATC 63

JAZF1 238 GCTCCACTGCACTCCAGGCTGGGTA--ACAGAGTGATACTTCATCTCAAATAAATAAATA 295

|| || || ||||||| ||||| | | |||| || ||||| ||||||| ||| ||| |

SUZ12 62 GCGTCATTGTACTCCAGCCTGGGCAATAAAGAGCGAAACTTCGTCTCAAAAAAAAAAAAA 3

JAZF1 296 AA 297

||

SUZ12 2 AA 1

**Genomic stem -8**

| **Score** | **Expect** | **Identities** | **Gaps** | **Strand** |
| --- | --- | --- | --- | --- |
| 159 bits(175) | 2e-43 | 221/306(72%) | 18/306(5%) | Plus/Minus |

JAZF1 1 GGCCAGGCACAGTGGCTCACGCCTATAATCCCAACAATTTGGGAGGCCAAGGCAGGTGAG 60

|||||||| |||||||||| |||| || ||||| || ||||||||||||||| ||

SUZ12 294 GGCCAGGCGCAGTGGCTCATGCCTGTATTCCCAGCACTTTGGGAGGCCAAGGTGGG---- 239

JAZF1 61 GCCTGAGGCACTTGAGCCCAGGAGTTCAGGACCAGCCACGGTAACACAGGGGGACCCTGT 120

| || ||| | || | ||||||| |||||||| |||| | | |||||||

SUZ12 238 --CAGAT-CACCTAAGGTCGGGAGTTCGAGACCAGCCTGACCAACATGGAGAAACCCTGT 182

JAZF1 121 CTCTGC-AAATAACTGTTTTTAAATTAGCCAGGTGCAGTGGCATG---TGCCTGTGGTCC 176

||| | ||||| | ||| ||||||| || ||| ||||||| |||

SUZ12 181 CTCCACCAAATA-----TACAAAAACAGCCAGGCATAGGGGCGGCGCATGCCTGTAATCC 127

JAZF1 177 TAGCTACTCAGGAGGCTGAGGCAGGAGGATCACCTGAGCCTGGGAGATTGAGGCTGCAGT 236

||||||| ||||||||||||||||| ||||| ||| |||||||| |||||||| ||

SUZ12 126 CAGCTACTTGGGAGGCTGAGGCAGGAGAATCACTTGAACCTGGGAGGCGGAGGCTGCCGT 67

JAZF1 237 GAGCCTAGATTGCACCACTGCACTTCAGCCTGGGCA--ACAGAGCAAGACCCTGTCTCAA 294

||||| |||| || || || ||| ||||||||||| | ||||| | || |||||||

SUZ12 66 GAGCCGAGATCGCGTCATTGTACTCCAGCCTGGGCAATAAAGAGCGAAACTTCGTCTCAA 7

JAZF1 295 AACAAA 300

|| |||

SUZ12 6 AAAAAA 1

**Genomic stem -11**

| **Score** | **Expect** | **Identities** | **Gaps** | **Strand** |
| --- | --- | --- | --- | --- |
| 80.6 bits(88) | 7e-21 | 96/126(76%) | 6/126(4%) | Plus/Minus |

JAZF1 1 TTTTTGTATTTTTAGTAGAGACGGGGTTTCACCATGTTGGCCAGGCTGGTCTCAAACTCC 60

||||| | ||||| | || | ||||| | |||||| |||||||||||||||||||

SUZ12 123 TTTTTTTCTTTTT--TTGAAAAGGGGTCTTGATATGTTGCCCAGGCTGGTCTCAAACTCG 66

JAZF1 61 TGACCTC--GTGATCCACCCGCCTCGGCCTCCCAAAGT-GCTGGGATTACAGGCGTGAGC 117

|| ||| ||||| || ||||| |||| ||| ||| ||| |||||||||| | || |

SUZ12 65 TGGGCTCAACAGATCCTCCTGCCTCAGCCT-CCAGAGTAGCTAGGATTACAGGTGGGATC 7

JAZF1 118 CACTGC 123

||||||

SUZ12 6 CACTGC 1

**Genomic stem -14**

| **Score** | **Expect** | **Identities** | **Gaps** | **Strand** |
| --- | --- | --- | --- | --- |
| 306 bits(339) | 5e-88 | 228/265(86%) | 4/265(1%) | Plus/Minus |

JAZF1 1 CCCAGGCTGGGGTGCAATGGCACGATCTTGGCTCACTGCAACCTCCACCTCCTGGGTTCA 60

|||||||||| || ||||| | ||||| ||||||||||||||||||||||| |||||||

SUZ12 263 CCCAGGCTGGAGTACAATGACGTGATCTCGGCTCACTGCAACCTCCACCTCCCGGGTTCA 204

JAZF1 61 AGCAATTCTCCTCCCTCAGCTTCCTGAGTAGCTGGGATTACAGATGCGCACCACCACACC 120

||||||||||| ||||||| ||| |||||||||||| ||||| || |||||||||| ||

SUZ12 203 GGCAATTCTCCTGCCTCAGCCTCCCGAGTAGCTGGGACTACAGGTGTGCACCACCACGCC 144

JAZF1 121 TGGCTAATTTTTGTATTTTTAGTAGAGACAGGGTTTCACCATGTTGGTCAGGCTGGTCTT 180

||||||||||||| ||||| |||||||||||||||||||||||||||||||||||||||

SUZ12 143 TGGCTAATTTTTGCATTTT--GTAGAGACAGGGTTTCACCATGTTGGTCAGGCTGGTCTT 86

JAZF1 181 GAACTCCTGACCTC--TTGATCCACCCACTTCAGCCTCCCAAAGTGCTGGGATTACAGGC 238

|||||||| ||||| ||||| | |||| | | ||||||||||||| |||||| |||

SUZ12 85 GAACTCCTCACCTCAGGTGATCTATCCACCCCTGTCTCCCAAAGTGCTAGGATTAAAGGT 26

JAZF1 239 ATGAGCCACTGCACCCGGCCAAAAA 263

|||||||| || |||||||||

SUZ12 25 GTGAGCCACCATGCCTGGCCAAAAA 1

**Genomic stem -31**

| **Score** | **Expect** | **Identities** | **Gaps** | **Strand** |
| --- | --- | --- | --- | --- |
| 126 bits(139) | 1e-33 | 221/317(70%) | 25/317(7%) | Plus/Minus |

JAZF1 1 GGCCAGGCACCGTGGCTCATACCTGTAATCCCAGCACTTGTG-AGGCTCTGG---GTGGA 56

|||| ||| | |||||||| ||||||||||||||||| | |||| || |||||

SUZ12 314 GGCCGGGCGCTGTGGCTCAGGTCTGTAATCCCAGCACTTCAGGAGGCCACGGTGAGTGGA 255

JAZF1 57 TCACTTGAGC--------------CCAGCCTGGGCAACATGGCAAACCCTTTCTCTACAT 102

|||| |||| |||||| || ||| |||| ||| || || |||

SUZ12 254 TCACCTGAGGTCAGGAGTTCAAGACCAGCCAGGCCAAAATGGTAAAACCCCAA-CTTCAT 196

JAZF1 103 GAAA--TACAAAAA-TTAGCTGGGTGTGATGGCATGTGCCTATAGTCCCAACTGCTCAGA 159

|||| |||||||| ||||| || ||| ||| | ||| || || || || | |

SUZ12 195 GAAAAATACAAAAAATTAGCCAGGCGTGGTGGTGGGCACCTGTAATCTCAGCTACCTGGG 136

JAZF1 160 AACCTGAGGTGGGAGGATCACCTGAGTCTGGGAGGTCAAGGCTACAGTAAGCCATGATGG 219

|| ||||| ||| ||| | ||| | | ||||| ||| | |||| ||||| ||| |

SUZ12 135 AAGCTGAGACAGGATAATCCCTTGAACCCGCGAGGTGGAGGTTGCAGTGAGCCAAGATTG 76

JAZF1 220 CACCACTGCACTCCAGCCTGGGTGAC-AGAGTAAGACCCTGTCTCAGaaaaaaaaagaaa 278

| ||| |||||||||||||||| || ||||| | || ||||||| ||||||||| |||

SUZ12 75 CGCCATTGCACTCCAGCCTGGGCAACAAGAGTGAAACTCTGTCTC--AAAAAAAAATAAA 18

JAZF1 279 aaaaaaagaaaagaaaa 295

|| |||| |||| ||||

SUZ12 17 AACAAAACAAAAAAAAA 1

**Genomic stem -36**

| **Score** | **Expect** | **Identities** | **Gaps** | **Strand** |
| --- | --- | --- | --- | --- |
| 74.3 bits(81) | 3e-18 | 146/211(69%) | 14/211(6%) | Plus/Minus |

JAZF1 1 GAAAACAAGAAAAAATTTAGCCAGGTACAGTGGCACATGCCTGTAGTC--AGCTATTTCA 58

||||| | |||||||| ||||||| |||| |||||| || ||||| |

SUZ12 202 GAAAAATACAAAAAATT-AGCCAGGCGTGGTGGTGGGCACCTGTAATCTCAGCTACCTGG 144

JAZF1 59 GAGGCTGAGGCAGGAGGATCACTTCAGCCCAAGAGGTCGAGGTCAAGGCTGCAGTGAACT 118

|| |||||| ||||| ||| ||| | ||| ||||| ||||| |||||||| |

SUZ12 143 GAAGCTGAGACAGGATAATCCCTTGAACCCGCGAGGTGGAGGT------TGCAGTGAGCC 90

JAZF1 119 ACGATCAAGCCACT-CACTCCAGCCTGG--AAGATGGCAGGGAGATGCTGTCTTTAAGaa 175

| ||| |||| | ||||||||||||| || | | || || | |||||| || ||

SUZ12 89 AAGATTGCGCCATTGCACTCCAGCCTGGGCAACAAG--AGTGAAACTCTGTCTCAAAAAA 32

JAZF1 176 aaaaaaaaaaaaaaaaaaaaGCAGAACAAAG 206

||| ||||| |||| ||||| | | |||||

SUZ12 31 AAATAAAAACAAAACAAAAAAAAAACCAAAG 1

**Genomic stem -38**

| **Score** | **Expect** | **Identities** | **Gaps** | **Strand** |
| --- | --- | --- | --- | --- |
| 36.2 bits(18) | 3e-09 | 18/18(100%) | 0/18(0%) | Plus/Minus |

JAZF1 1 CACGCCTGTAATCCCAGC 18

||||||||||||||||||

SUZ12 18 CACGCCTGTAATCCCAGC 1

**Genomic stem -39**

| **Score** | **Expect** | **Identities** | **Gaps** | **Strand** |
| --- | --- | --- | --- | --- |
| 30.2 bits(15) | 1e-07 | 15/15(100%) | 0/15(0%) | Plus/Minus |

JAZF1 1 TGTTGCCCAGGCTGG 15

|||||||||||||||

SUZ12 15 TGTTGCCCAGGCTGG 1

**Genomic stem -44**

| **Score** | **Expect** | **Identities** | **Gaps** | **Strand** |
| --- | --- | --- | --- | --- |
| 28.2 bits(14) | 4e-07 | 14/14(100%) | 0/14(0%) | Plus/Minus |

JAZF1 1 GGGAAAAAGAAACA 14

||||||||||||||

SUZ12 14 GGGAAAAAGAAACA 1

**Genomic stem -45**

| **Score** | **Expect** | **Identities** | **Gaps** | **Strand** |
| --- | --- | --- | --- | --- |
| 28.2 bits(14) | 4e-07 | 14/14(100%) | 0/14(0%) | Plus/Minus |

JAZF1 1 TCAACTTTAAATGT 14

||||||||||||||

SUZ12 14 TCAACTTTAAATGT 1

**Genomic stem -51**

| **Score** | **Expect** | **Identities** | **Gaps** | **Strand** |
| --- | --- | --- | --- | --- |
| 28.2 bits(14) | 7e-07 | 17/18(94%) | 0/18(0%) | Plus/Minus |

JAZF1 1 AAAAAGTGAAATAACCCA 18

|||||| |||||||||||

SUZ12 18 AAAAAGAGAAATAACCCA 1

**Genomic stem -53**

|  | | | | |
| --- | --- | --- | --- | --- |
| **Score** | **Expect** | **Identities** | **Gaps** | **Strand** |
| 25.6 bits(27) | 2e-06 | 15/16(94%) | 0/16(0%) | Plus/Minus |

JAZF1 1 ATAAAAAATGCTTTGA 16

|| |||||||||||||

SUZ12 16 ATTAAAAATGCTTTGA 1
