# Supplementary material for: RNA-driven JAZF1-SUZ12 gene fusion in human endometrial stromal cells
Source: PLoS Genet. 2021 Dec 20;17(12):e1009985. doi: 10.1371/journal.pgen.1009985 (PMC8722726; doi:10.1371/journal.pgen.1009985)
Supplement: S4 Text — (DOCX) [file pgen.1009985.s012.docx]

**S4 Text. Genomic coordinates (UCSC Blat-hg38) for JAZF1 and SUZ12 sequences constituting the genomic stems.**

|  | **JAZF1** | **SUZ12** |
| --- | --- | --- |
| **Genomic stem -1** | 27850950 - 27851231 | 31939530 - 31939814 |
| **Genomic stem -2** | 27887419 - 27887715 | 31939525 - 31939825 |
| **Genomic stem -8** | 27880675 - 27880974 | 31939532 - 31939825 |
| **Genomic stem -11** | 27845425 - 27845547 | 31939291 - 31939413 |
| **Genomic stem -14** | 27815749 - 27816011 | 31946247 - 31946509 |
| **Genomic stem -31** | 27813341 - 27813635 | 31942323 - 31942636 |
| **Genomic stem -34** | 27821423 - 27821650 | 31944859 - 31945077 |
| **Genomic stem -36** | 27813135 - 27813340 | 31942316 - 31942517 |
| **Genomic stem -38** | 27887681 - 27887698 | 31943828 - 31943845 |
| **Genomic stem -44** | 27815392 - 27815405 | 31947564 - 31947577 |
| **Genomic stem -45** | 27819520 - 27819533 | 31947621 - 31947634 |
| **Genomic stem -51** | 27822270 - 27822287 | 31941089 - 31941106 |
| **Genomic stem -53** | 27831391 - 27831406 | 31943997 - 31944012 |
